# Comprehensive review of sweetpotato flavor compounds: Opportunities for developing consumer‐preferred varieties

**DOI:** 10.1111/1541-4337.70172

**Published:** 2025-04-24

**Authors:** Modesta Abugu, Matthew Allan, Suzanne Johanningsmeier, Massimo Iorizzo, G. Craig Yencho

**Affiliations:** ^1^ Department of Horticultural Science North Carolina State University Raleigh North Carolina USA; ^2^ Food Science and Market Quality & Handling Research Unit United States Department of Agriculture, Agricultural Research Service Raleigh North Carolina USA; ^3^ Plants for Human Health Institute, Department of Horticultural Science North Carolina State University Kannapolis North Carolina USA

**Keywords:** *Ipomoea batatas*, Maillard reaction, sensory evaluation, Sweet potato, volatile organic compounds

## Abstract

Flavor contributes significantly to consumer preferences of cooked sweetpotato. Sugars largely drive the sweet taste, while the volatile organic compounds (VOCs), mainly classified as alcohols, aldehydes, ketones, and terpenes, provide characteristic aromas and influence the overall perception of flavor. In this paper, we review sweetpotato VOCs identified in the literature from 1980 to 2024 and discuss the efforts to understand how these compounds influence sensory perception and consumer preferences. Over 400 VOCs have been identified in cooked sweetpotato with 76 known to be aroma‐active. Most of these aroma‐active compounds are generated from Maillard reactions, Strecker, lipid and carotenoid degradation, or thermal release of terpenes from glycosidic bonds during cooking. Suggested mechanisms of formation of these aroma‐active compounds are described. However, specific VOCs that are responsible for different aromas and flavors in cooked sweetpotatoes are yet to be fully characterized. There are significant opportunities to further identify the key predictors of aroma and flavor attributes in sweetpotato, which can be used to enhance the quality of existing varieties and develop new ones using a wide range of genetic tools. This review summarizes 44 years of research aimed at identifying key aroma compounds in cooked sweetpotato and provides a roadmap for future studies to guide breeders in developing high‐quality, consumer‐preferred varieties.

## 1 INTRODUCTION

Sweetpotato production in the United States has nearly tripled over the past two decades, with fresh market production accounting for about $700 million of the farm‐gate value (USDA‐NASS, 2022). This growth can be attributed to the high nutritional properties of the crop and consumers’ increasing demand for healthier food options (Truong et al., 2018). In addition to carbohydrates, proteins, and lipids that are present in sweetpotato (Cartier et al., 2017; Drapal et al., 2019; Wang et al., 2016), the orange‐fleshed and purple‐fleshed varieties have significant levels of β‐carotene and anthocyanins, two pigments that are associated with numerous health benefits (Grace et al., 2014; Sosa et al., 2024; Wang et al., 2016). The versatility of sweetpotato, its multipurpose use in a wide range of value‐added products, vibrant colors that enhance mealtimes, and its potential to help prevent age‐related illnesses have all contributed to increased consumer awareness of its benefits (George et al., 2024).

Sweetpotatoes are prepared for consumption in various ways, such as baking, frying, roasting, steaming, boiling, and microwaving, depending on the region where they are consumed (Wang et al., 2016). In the United States, baking is a common cooking method (Wang & Kays, 2000) and is preferred by consumers over microwave cooking (Barkley et al., 2017). The cooking process facilitates the breakdown of sweetpotato metabolites, resulting in the formation of unique flavors, which are major determinants of consumer preference and overall liking (Chan et al., 2014; Leksrisompong et al., 2012).

The flavor of cooked sweetpotato is influenced by a complex balance of taste and aroma attributes (Wang & Kays, 2000). Tastes (sweet, salty, sour, bitter, and umami) are detected by the tongue, whereas aromas are a response from volatile organic compounds (VOCs) stimulating olfactory receptors in the nasal cavity before (orthonasal), during (retronasal), and after consumption (Goldberg et al., 2017). The perceived flavor of foods can also be influenced by multi‐modal interactions and consumer expectations (Diamond et al., 2005; Piqueras‐Fiszman & Spence, 2015), but these effects have not been well studied in sweetpotato. Here, we focus on VOCs as the physiological stimuli detected through ortho‐ and retronasal olfaction and identified by different targeted and non‐targeted methods in cooked sweetpotato.

Gas chromatography and mass spectrometry (GC‐MS) instrumental methods have been widely applied in identifying VOCs in sweetpotato. GC‐MS enables the identification of a multitude of compounds; however, associating these to aroma and flavor perception requires additional instrumental (i.e., GC‐olfactometry—GC‐O) and/or sensory evaluation techniques. For example, the use of aroma activity values, including aroma extract dilution analysis (AEDA), flavor dilution (FD) value, or relative odor activity value have been utilized to identify aroma‐active compounds in cooked sweetpotato (Jiang et al., 2023; Kays & Wang, 2003; Nakamura et al., 2013; Shen et al., 2024; Sun et al., 1995; Tiu et al., 1985; Wang & Kays, 2000). Using the AEDA method, Sun et al. (1995) identified maltol as an important aroma‐active compound for baked sweetpotato aroma. More recently, Shen et al. (2024) used GC‐O and descriptive sensory analysis to correlate VOCs with sweetpotato sensory attributes. Furthermore, Hou et al. (2020) identified one compound (2‐furanmethanol) that was associated with the overall acceptability of roasted sweetpotato. However, since flavor is a complex sensation derived from simultaneous perception of many VOCs, one compound is likely insufficient to broadly predict acceptance or liking of cooked sweetpotato.

Current studies on sweetpotato did not factor in the interaction effects among VOCs on flavor attributes. Different compounds can influence the detectability of individual odorants, potentially skewing their aroma activity values (Regueiro et al., 2017). Studies in other foods described these effects as either synergistic, additive, or masking (Chen et al., 2020). For example, the fruity aroma in liquors was masked when various levels of ethyl phenylacetate were added, while the floral note was enhanced (synergistic effect) at low or high levels (Niu et al., 2018). There is still much to learn about the VOCs that influence specific flavors, the intricate relationships among different VOCs, and the impact of these on cooked sweetpotato flavor profiles.

In addition to determining the important predictors of flavor in cooked sweetpotato, understanding the mechanism of formation of those VOCs would aid the efficient development of sweetpotato varieties that meet consumer preferences. Previous studies have suggested different mechanisms of formation of VOCs in sweetpotato and model systems (Hidalgo et al., 2013; Sun et al., 1995; Tsai et al., 2021; Wang & Kays, 2000). Overall, sweetpotato VOCs are derived from carbohydrates, amino acids, lipids, β‐carotene, and storage forms of terpenoid precursors through various thermal reactions (Sun et al., 1995; Tsai et al., 2021; Wang & Kays, 2000; Yao et al., 2024). Several excellent review articles have described the composition of potential VOC precursors in different sweetpotato cultivars (Abbas et al., 2023; De Albuquerque et al., 2019; Drapal et al., 2019; Laveriano‐Santos et al., 2022; Truong et al., 2018); however, to our knowledge, there has been no comprehensive review on VOC formation in cooked sweetpotato.

Therefore, this review describes the existing studies on the composition of sweetpotato VOCs identified from 1980 to 2024, the mechanisms of formation of select VOCs, and the contributions of VOCs to different aroma and flavor profiles in sweetpotato. Furthermore, the instrumental and sensory tools utilized in sweetpotato flavor detection are discussed, as well as factors that influence VOCs composition in cooked sweetpotato. Finally, opportunities for developing high‐quality varieties by integrating novel genetic tools and a food science approach are discussed.

## 2 METHODS

### 2.1 Literature search

We conducted a broad search in three databases (Web of Science, CABAbstracts, and Food Science and Technology Abstracts—FSTA) that publish peer‐reviewed food flavor research in horticultural crops, using the EBSCO information services research database (EBSCO). The search was conducted using key words, including combinations of sweet potato, *Ipomoea batatas*, sweetpotato, flavor or flavour, VOCs, sugars, flavor chemistry or flavour chemistry, flavour profiles or flavor profiles, organoleptic compounds, sweetness, and aroma. To further ensure that no articles were missing, additional key words such as baked sweetpotato, roasted sweetpotato, boiled sweetpotato, GC, MS, or GC‐MS were manually keyed into Google Scholar and the results compared to the outputs from the database search. The complete strategy for the literature search is available in the Supporting Information (Table S1).

### 2.2 Study selection

**FIGURE 1:**
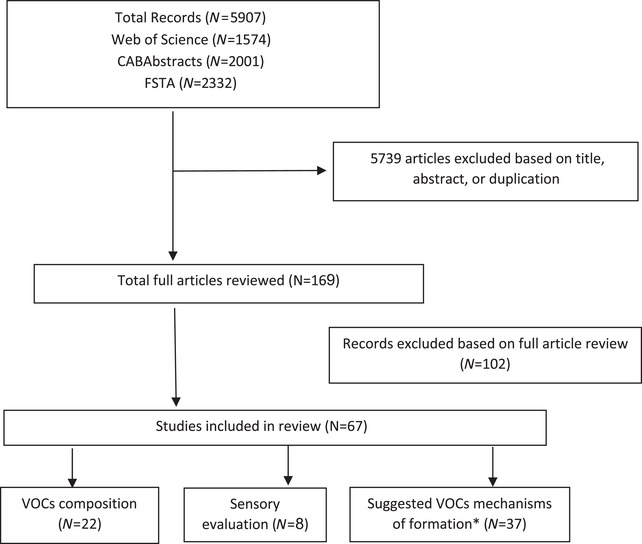
Studies screened and selected for inclusion in the review of volatile compositions of cookedsweetpotato. *indicates articles discussing VOCs formation in other crops. FSTA, Food Science and Technology Abstracts; VOCs, volatile organic compounds.

The filtering strategy used to include and exclude articles for the review is described in Figure 1. During the initial search, a total of 1574, 2001, and 2332 articles were found for the Web of Science, CABAbstracts, and FSTA databases, respectively. After exclusion of articles (*n *= 5739) based on title, abstract, or duplication, a total of 169 articles remained. The full text of these articles were reviewed, and 67 articles including those pertaining to sweetpotato flavor as identified using instrumental techniques and different cooking methods (*n* = 22) were retained and used as primary articles. Those pertaining to sensory evaluation techniques and consumer perception (*n* = 8), and those that suggested mechanism of formation of VOCs in sweetpotato model systems and other crops (*n* = 37), were also included and used as supplemental articles. Studies outside the scope of this review (*n* = 102) were removed, including articles discussing taste, non‐volatile acids and sugars in sweetpotato, storage mechanism, volatile compounds released due to damage or disease infestation, nutritional and quality studies, gender‐based studies, and phenotyping methods for sweetpotato texture. A list of all included and excluded articles, and reasons for exclusion is described in the Supporting Information (Section 1D).

## 3 RESULTS

This review discusses findings that are based on 67 highly relevant publications, including 22 fundamental research articles identifying VOCs in cooked sweetpotatoes and 45 supporting articles. The supporting articles covered topics such as (1) mechanisms of VOC formation as demonstrated in other crops or model systems, (2) instrumental identification techniques of VOCs in foods, (3) sensory evaluation techniques for identifying VOCs that influence flavors, and (4) opportunities for using genetic tools and food science in developing new varieties with distinct flavor profiles. This first and only comprehensive review of sweetpotato VOC composition brings together information on the complex nature of sweetpotato flavor, informing breeding strategies for the development of consumer‐preferred varieties.

### 3.1 VOCs that contribute to known aroma profiles of sweetpotato vary and are not fully characterized

**FIGURE 2:**
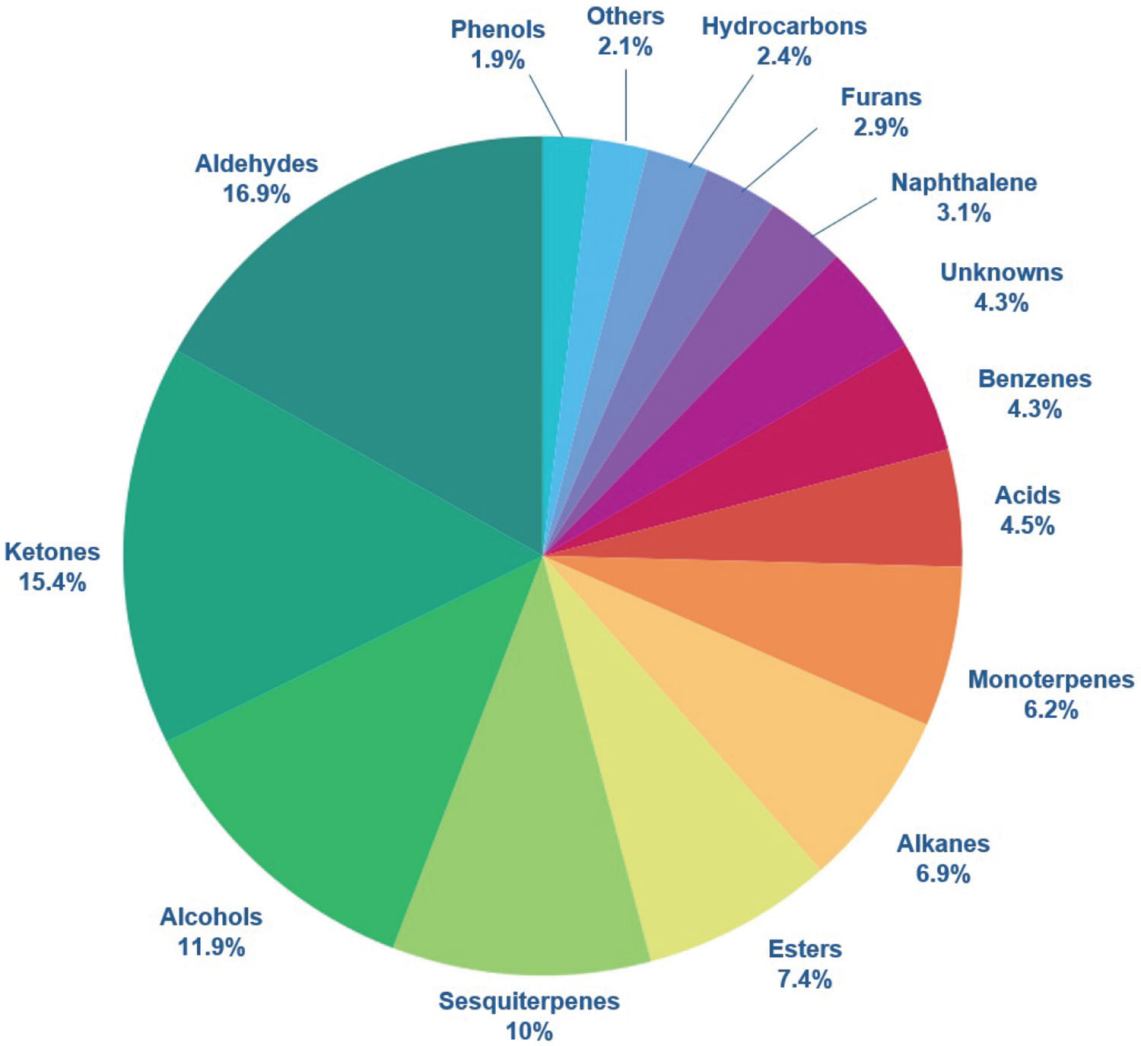
Classification of all groups of volatile compounds identified in cooked sweetpotato from 1980s to present. See Supporting Information Table S2 for the list of all compounds.

Over 400 unique VOCs have been identified in cooked sweetpotato (Table S2) across 22 studies investigating sweetpotato aroma compounds with various GC‐MS methods. Aldehydes, ketones, alcohols, and sesquiterpenes were the dominant VOCs, comprising about 17%, 15%, 12%, and 10% of the total VOC composition, respectively (Figure 2). Other notable compound groups include esters (7%), alkanes (7%), monoterpenes (6%), acids (5%), benzenes (4.3%), naphthalenes (3.1%), phenols (1.9%), hydrocarbons (2.4%), and unknowns (4.3%). It must also be noted that a single VOC does not represent the full aroma of cooked sweetpotato; rather, sweetpotato aroma is influenced by a complex balance of many compounds (Wang & Kays, 2000). Sweetpotato genotype, flesh color, and cooking method impact the ratios of the VOC groups, and understanding the contributions of these compounds to the aroma and flavor of cooked roots is still an active area of research (Shen et al., 2024; Sun et al., 1995; Wang & Kays, 2001; Yao et al., 2024; Zhang et al., 2021). Of over 400 compounds identified, 76 are thought to be aroma‐active (Table S3). The suggested mechanisms of formation of some of these compounds are presented in Table 1. Ten of these aroma‐active VOCs are thought to be derived from carbohydrate degradation during Maillard reaction (MR); five are thought to be derived from Strecker degradation (SD) of amino acids during MR; 12 are thought to come from lipid degradation; seven are known to come from carotenoid degradation; and 15 are thought to come from the thermal release of glycosidically bound terpenes. Some other VOCs (*n* = 6) are suggested to be derived from degradation mechanisms that are unknown (Table 1). A summary of these suggested mechanisms is detailed below.

**TABLE 1:** Suggested mechanisms of formation of aroma‐active volatile organic compounds (VOCs) identified in cooked sweetpotatoes.

Suggested mechanism of formation	Aroma‐active VOCs	Odor descriptors	References
Maillard reaction/caramelization of carbohydrates	maltol, furneol 2‐acetyl pyrrole 2‐furanmethanol, acetol 2‐acetyl‐furan, 3‐furaldehyde, 5‐methylfurfural, 5‐hydroxymethylfurfural, furfuryl alcohol	Caramel*^z^, sweet, caramel‐like*, sweet/burnt flavors*, caramel‐like*^z^, coconut^f^, baked potato*, honey^f^, burnt, sweet, caramel*^z^, bread, almond, sweet^f^, burnt^f^	Wang and Kays (2000) Wang and Kays (2003) Nakamura et al. (2013) Shen et al. (2024)
Strecker degradation of amino acids	phhenylacetaldehyde, benzaldehyde, methional, phenethyl alcohol, benzyl alcohol	Sweet, perfume*, burnt sugar, almond*^z^, boiled potato flavor^f^, honey, spice, lilac^f^, sweet, flower^f^	Wang and Kays (2000) Nakamura et al. (2013) Jiang et al. (2023) Shen et al. (2024) Yao et al. (2024)
Lipid oxidation	hexanal, decanal, nonanal, octanal, n‐decanal, 2,4‐decadienal, 2,4‐nonadienal, 2‐pentyl‐furan, 2‐octenal, (E)‐2‐nonenal, (E,E)‐2,4‐heptadienal, (E,E)‐2,4‐nonadien‐1‐al	Grass*, tallow*, green, grassy*, oily*^z^, green*^z^, green, fatty*, musty, cooked, green and fatty*, floral, nutty^f^, green and fatty^f^, fat, soap, lemon green^f^, floral, oily*^z^, fried^f^, cucumber, green^f^	Wang and Kays (2003) Jiang et al. (2023) Yao et al. (2024)
Thermal release of glycosidically bound terpenes	**Monoterpenes** methyl geranate, geraniol, linalool, nerol, α‐terpinene, myrtenol, citronellol, **Sesquiterpenes** cyperene, α‐copaene, allo‐aromadendrene, p‐cymen‐8‐ol, cedrol, cuminal, dehydro‐1,8‐cineole, 4‐hydroxy‐3‐methoxystyrene	Sweet candy*, sweet floral^f^, sweet flower*, citrus*^z^, sweet*, fruit, peachy‐like^f^, muscat flavor^f^, pine oil*^z^, earthy, baked potato*, hot apple, sweet^f^, balsamic odor^f^, clove, spice, sweet*^z^, acid, shar^f^, mint, lemon^f^, balsmic^f^	Wang and Kays (2000) Wang and Kays (2003) Tsai et al. (2021) Jiang et al. (2023) Yao et al. (2024) Shen et al. (2024)
Carotenoid degradation	β‐ionone dihydroactinidiolide, β‐cyclocitral, geranyl acetone, β‐damascenone, 5,6‐epoxy‐β‐ionone	Violet*, sweet*, floral^f^, tea‐like^f^, rose, floral, honey*^z^, fruity aroma^f^, fruit, seaweed, wood^f^	Wang and Kays (2000) Waché et al. (2003) Wang and Kays (2003) Jiang et al. (2023)
Unknown mechanism	guaiacol, p‐vinylguaiacol, eugenol, 2‐methoxy‐4‐vinylphenol acetic acid, dimethyl trisulfide	Burnt*^z^, earthy*^z^, clove, honey^f^, sulfur, fish, cabbage*^z^ clove, spice*^z^, intense violet aroma^f^	Y. Wang and Kays (2000) Y. Wang and Kays (2001) Tsai et al. (2021) Jiang et al. (2023) Shen et al. (2024)

*Indicates odors that have been characterized in cooked sweetpotato using gas chromatography‐olfactometry (GC‐O).

*^z^ Indicates odor characterized by GC‐O and sensory analysis.

^f^Aroma characteristics from Flavornet, ^https://www.flavornet.org/flavornet.html.^

### 3.2 Mechanisms of formation of sweetpotato VOCs

#### 3.2.1 The Maillard Reaction (MR) generates VOCs through a complex cascade of reactions

The MR is a complex series of non‐enzymatic chemical reactions that occur between carbonyl and amine compounds. In foods, MR commonly occurs between reducing sugars and amino acids during cooking, but the carbonyl‐containing compound may also come from lipid oxidation or MR products. The MR can generate hundreds to thousands of volatile and non‐volatile compounds, whereas non‐MR degradation products of isolated amino acids and sugars are substantially fewer (Hemmler et al., 2017). These MR products play important roles in enhancing the sensory characteristics of food by contributing color, aroma, and taste‐active compounds (Damodaran & Parkin, 2017). It is also believed that the MR products are the most abundant source of VOCs that have been identified in cooked sweetpotato (Shen et al., 2024; Sun et al., 1995; Tsai et al., 2021; Wang & Kays, 2000). These products, which include aldehydes, esters, furans, ketones, and pyrazines were reported to impart a wide range of aromas in cooked sweetpotato through aroma activity studies (Table 1).

**TABLE 2:** VOCs identified in cooked sweetpotatoes from 1980‐2024, arranged by year of article publication. The methods used for cooking, extraction and identification are also shown.

Year published	Cooking method	Extraction method	Identification method	No of VOCs identified	No of aroma‐active VOCs	References
1980	Baking	Purge and trap	GC‐mass spectrometry (GC‐MS)	30	nd	Purcell et al. (1980)
1985	Baking	Purge and trap	GC‐MS, GC‐O	27	13^a^	Tiu et al. (1985)
1991	Baking	Steam distillation	GC‐MS	21	nd	Horvat et al. (1991)
1993	Baking	Purge and trap	GC‐MS	23	nd	Sun et al. (1993)
1994	Microwaving	Purge and trap	GC‐MS	13	nd	Sun et al. (1994)
1995	Baking	Purge and trap	GC‐MS	14	1^c^	Sun et al. (1995)
1998	Baking	Purge and trap	GC‐MS	46	38^a^	Wang et al. (1998)
2000	Boiling	Purge and trap	GC‐MS	6	nd	Kays and Wang (2000)
2000	Baking	Purge and trap	GC‐flame‐ionized detector (GC‐FID)	60	37^a^	Wang and Kays (2000)
2001	Baking, boiling, microwaving	Purge and trap	GC‐MS, GC‐O	37	21^a^	Wang and Kays (2001)
2003	Baking	Headspace solid‐phase microextraction (HS‐ SPME)	GC‐FID, GC‐O	19	17^a^	Kays and Wang (2003)
2005	Baking	Purge and trap	GCMS	16	nd	Dumas et al. (2005)
2013	Boiling	Hydro distillation	GC‐MS, GC‐O	75	18^a^ ^e^	Nakamura et al. (2013)
2020	Roasting	HS‐ SPME	GC‐MS	62	1^e^	Hou et al. (2020)
2021	Boiling	HS‐ SPME	GC‐FID	40	nd	Ravi et al. (2022)
2021	Roasting	HS‐SPME	GC‐MS	46	3^e^	Tsai et al. (2021)
2021	Baking	HS‐SPME	GC‐MS	68	nd	Zhang et al. (2021)
2023	Baking, steaming boiling	HS‐SPME	GC‐MS	42	nd	Yao et al. (2023)
2023	Baking, steaming, boiling	HS‐SPME	GC‐MS	64	22 ^c^	Jiang et al. (2023)
2023	Steaming	HS‐SPME	GC‐MS	57	nd	Yao et al. (2024)
2023	Baking, steaming, boiling	HS‐SPME	GC‐MS	72	nd	Zhang et al. (2023)
2024	Roasting	HS‐SPME	GC×GC‐ToFMS, GC‐O	120	18^a^, ^c^	Shen et al. (2024)

Abbreviation: nd, not determined; ToFMS, time‐of‐flight mass spectrometer.

^a^ Aroma‐active as reported in cooked sweetpotatoes using either GC‐O or aroma extract dilution analysis calculation methods.

^b^ Relative odor activity value calculation method through comparison to odor threshold studies in the literature (Yao et al., 2024).

^c^ Trained descriptive sensory evaluation.

^d^ informal sensory evaluation.

**FIGURE 3:**
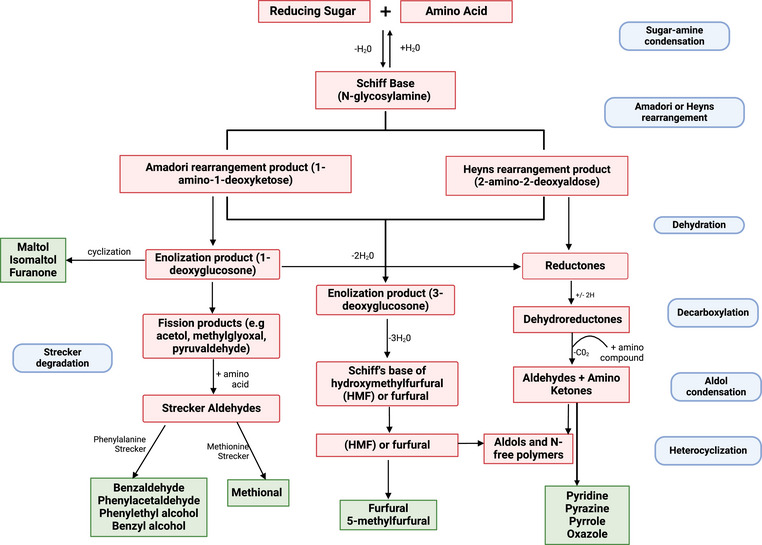
Schematic representation of the reactions involved in the synthesis of VOCs derived from Maillard reaction and Strecker degradation of amino acids. Light red‐shaded boxes indicate the precursors and intermediates; light green‐shaded boxes are the VOCs formed from the different reactions that have been identified in cooked sweetpotato; and light blue‐shaded boxes are names of reactions. Mechanism modified from Varlet et al. (2007) and Feng et al. (2022).

The MR pathway that leads to the formation of VOCs in foods is highly complex and has been reviewed by numerous authors (Habinshuti et al., 2021; Hemmler et al., 2017; Liu et al., 2022; Parker, 2015). Briefly, the carbonyl of a reducing sugar in the temporary open ring structure undergoes a nucleophilic attack from an unprotonated primary amine of an amino acid or peptide. This condensation reaction produces a Schiff base that can cyclize into a N‐substituted glycosylamine (Figure 3). These compounds then undergo reversible Amadori rearrangements if the sugar was an aldose (e.g., glucose and maltose) or Heyns rearrangements with a ketose (e.g., fructose) and can result in new carbonyl (i.e., ketone) containing compounds (e.g., 1‐amino‐1‐deoxy‐2‐ketose). With continued heating, these products can form new Schiff bases with amino acids or undergo additional dehydration reactions followed by a cascade of complex reactions. The dehydration, condensation, fission, cyclization, SD (reaction between an MR dicarbonyl and amino acid), and other reactions of intermediates result in a vast array of VOCs (Martins et al., 2000; Van Boekel, 2006). For example, a simple histidine + ribose reaction at 100°C for 10 h, resulted in 348 detected compounds (Hemmler et al., 2017). In foods, the MR is even more complex because there are multiple types of reducing sugars and the amine can be from any of the 20+ free amino acids or a peptide. Therefore, we will further discuss the impact of the types of carbohydrates and amino acids in the MR on the VOCs in cooked sweetpotatoes.

#### 3.2.2 Sweetpotato carbohydrates and the impact on VOCs

Raw sweetpotatoes are relatively high in sugars, compared to other root and tuber crops. The most common orange‐fleshed sweetpotato (OFSP) varieties in the United States, Covington or Beauregard, generally contain about 1% glucose, 1% fructose, and 2% sucrose as raw roots (Rolston et al., 1987; Yencho et al., 2008). Additional reducing sugars and starch‐reducing ends are generated when sweetpotatoes are cooked because semi‐heat stable amylases produce maltose and starch dextrins. The amount and extent of starch hydrolysis depend on overall amylase activities and the length of time in which starch is gelatinized before the amylases are thermally inactivated (Purcell & Walter, 1988). For example, a microwaved sweetpotato will generate less maltose than a baked sweetpotato (Barkley et al., 2017; Sun et al., 1994) because it is heated faster, resulting in less time between gelatinization and amylase denaturation. This temperature window is between the onset of sweetpotato starch gelatinization around 55 to 65°C (Allan et al., 2023) and the inactivation temperatures of sweetpotato α‐ and β‐amylases around 80 and 70°C, respectively (Hagenimana et al., 1994). During this time, α‐amylase hydrolyzes starch into dextrins at random α‐(1,4) linkages, and β‐amylase cleaves the dextrins from non‐reducing ends into maltose molecules. Therefore, this amylase activity greatly increases the number of reducing ends and is critical in generating MR substrates and thus VOCs.

Caramelization is another type of non‐enzymatic browning reaction of sugars that likely occurs along with the MR during sweetpotato cooking. All sugars can undergo caramelization, which occurs at high temperatures and only requires sugars. This caramelization process also generates brown pigments and VOCs similar to those generated during MR (Hemmler et al., 2018). Sucrose is the primary non‐reducing sugar in sweetpotato, and unless hydrolyzed or degraded into an aldehyde/ketone, it will not participate in the MR but could undergo caramelization. Depending on the time and temperature of cooking, caramelization could likely generate sweetpotato VOCs, which are difficult to distinguish from MR since the VOCs are similar.

Sun et al. (1995) provided one of the first pieces of evidence that the MR generates key sweetpotato VOCs. They identified several furan‐based VOCs that were present in both baked sweetpotato and cooked sugars. Furans (e.g., 5‐methylfurfural, 2‐furanmethanol, furfural) have also been reported as notable VOCs in cooked sweetpotato in other studies (Hou et al., 2020; Shen et al., 2024; Sun et al., 1995; Yao et al., 2023). Aroma activity analysis of furans using GC‐O showed these compounds imparted caramel‐like, sweet, baked potato, spicy‐sweet, and burnt aromas (Wang & Kays, 2001). Furans are thought to form from sugar degradation following several dehydration and decarboxylation reactions before cyclization into the five‐membered aromatic ring.Sun et al. (1995) also reported that maltol (pyran), another sugar breakdown product, was essential to the characteristic aroma of baked sweetpotato. Maltol has subsequently been reported as one of the top VOCs in roasted/baked sweetpotato in several studies (Hou et al., 2020; Shen et al., 2024; Tsai et al., 2021; Wang & Kays, 2001). Interestingly, little to no maltol forms via maltose caramelization nor in MR with monosaccharides (Kanzler et al., 2017; Yaylayan & Mandeville, 1994). Since sucrose cannot participate in the MR, this suggests maltose is the primary source for the generation of maltol in cooked sweetpotato, highlighting the impact of the starch to maltose conversion on VOC formation during cooking. In summary, carbohydrate breakdown products from the MR, and possibly caramelization, contribute to key cooked sweetpotato VOCs. Additional studies on thermal degradation, enzymatic conversions, kinetic modeling, and processing effects will aid further understanding of the formation of carbohydrate‐derived VOCs and uncover factors that may influence their generation in cooked sweetpotato.

#### 3.2.3 Amino acids and peptides and the impact on sweetpotato VOCs

Amino acids and peptides are capable of participating in the MR. However, only free amino acids and the terminal amine plus amine R‐groups (e.g., lysine) of a peptide can participate. It has been well demonstrated that the type of amino acid participating in the MR will impact the VOCs generated (Liu et al., 2022). For example, MR with phenylalanine + glucose smelt floral‐like, leucine + glucose smelt like burnt caramel, and cysteine + glucose smelt sulfury (Wong et al., 2008). In the MR pathway, an amino acid is needed to form the initial glycosylamine, but amino acids can also undergo SD with MR dicarbonyl compounds. SD generates Strecker aldehydes and 2‐aminocarbonyls through a transamination reaction and a series of decarboxylation and hydrolysis reactions (Figure 3). The final VOCs produced depend on the amino acid precursor (Yaylayan, 2003). For example, SD of α‐dicarbonyls with phenylalanine lead to the generation of phenylacetaldehyde/benzeneacetaldehyde (floral, sweet, and honey‐like aromas) and subsequently benzaldehyde (almond and cherry‐like aromas; Chu & Yaylayan, 2008; Hofmann & Schieberle, 2000), while SD of methionine leads to the generation of methional, which smells like cooked potato (Yaylayan & Keyhani, 2001). Further, leucine can be degraded to the intermediate 2‐isobutyl‐5‐methyl‐3‐oxazoline, which leads to 3‐methylbutanal, 2‐sec‐butyl‐5‐methyl‐3‐oxazoline, or 2‐methylbutanal that elicit malty, cocoa, or almond flavors (Fu et al., 2020). Threonine can also react with α‐dicarbonyls and undergo reductive amination to form 2‐amino ketones. The reactive primary amino group in the 2‐amino ketones then allows dimerization with itself or other reactive aldehydes or ketones to generate the N‐containing heterocyclic aroma compounds such as pyrazines, pyrroles, and oxazole compounds that impart roasted, earthy and spicy aromas in roasted nuts, cooked meat and coffee respectively (Fu et al., 2020; Liu et al., 2022; Yaylayan, 2003). Another VOC present in sweetpotato and formed from MR of threonine and serine is 2,3‐butanedione, a VOC that mostly imparts a buttery note in fried potatoes (Van Loon et al., 2005).

Evidence of the SD of amino acids during MR was demonstrated by Habinshut et al. (2021) using ultrasound‐assisted enzymatic treatment of sweetpotato protein hydrolysates using a xylose MR model system. The study indicated that crosslinking between sweetpotato peptides (SPPs) and xylose during MR and SD led to a significant decrease in both the free and total amino acid contents of SPPs, likely due to the initiation of VOC formation (Habinshuti et al., 2021). The authors further confirmed this by analyzing the profiles of the MR products formed from the enzymatic treatments and found that SPP showed higher levels of VOCs from SD. Among them were benzaldehyde and phenylacetaldehyde, known for their contributions to malty/rose, perfume, and floral aromas in model systems (Jiang et al., 2023; Shen et al., 2024).

Yao et al. (2024) identified a significant correlation between 12 amino acids and 11 VOCs in five sweetpotato cultivars such that the variety with high phenylalanine content also contained high phenylacetaldehyde (Yao et al., 2024); however, others reported a negative correlation between phenylacetaldehyde and phenylalanine (Hou et al., 2020). The differences in cooking methods used in the two papers (baking in vial vs. roasting) could explain the variation in correlation observed, as both authors utilized similar sample prep methods and instrumentation techniques to identify the amino acids and VOCs. In support of this hypothesis, Wang and Kays (2001) reported that the cooking cycle, moisture conditions, and method of heat introduction could change the VOC profile of sweetpotato since specific temperatures are needed for the sweetpotato metabolites to break down to VOC. Understanding how these factors influence overall flavor profile would be useful for a full appreciation of this mechanism in cooked sweetpotato.

Phenylalanine may not be the only amino acid involved in the formation of phenylacetaldehyde as other amino acids such as cysteine or serine are suggested to play a role in its formation. Hidalgo et al. (2013) reported over 600% and 500% increases in the amount of phenylacetaldehyde generated when phenylalanine was heated at 200°C for 1 h in the presence of cysteine and serine, respectively. Sweetpotato contains significant levels of phenylalanine and serine with relatively low levels of cysteine, depending on the genotypes evaluated (Drapal et al., 2019; Qiu et al., 2020; Yao et al., 2024). Therefore, further investigation into their degradation during cooking could help inform how these aromatic compounds are generated.

#### 3.2.4 Lipids and the impact on sweetpotato VOCs

Although relatively lower than other foods, sweetpotato storage roots contain some unsaturated fatty acids (polyunsaturated fatty acids [PUFAs]), with linoleic, linolenic, and oleic acids as the most abundant (Wang et al., 2016). Linoleic (C18:2) and linolenic (C18:3) acids are PUFAs with multiple double bonds in the hydrocarbon chain and are the most susceptible to oxidation. Monounsaturated fatty acids, such as oleic acid (C18:1), are less susceptible but still much more prone to oxidation than saturated fatty acids, which have no double bonds. PUFAs are thought to be the main precursors of some important lipid‐derived VOCs, including 2‐hexenal, nonanal, 2,4‐decadienal, 2,4‐ nonadienal, n‐decanal, 2‐pentenal, and heptanal, seven compounds that are abundant in cooked sweetpotato (Shen et al., 2024; Tsai et al., 2021; Yao et al., 2024). These VOCs are known to impart green leaf or fat‐like notes in fruits and vegetables (Shahidi & Hossain, 2022). Studies have shown that these compounds are either produced through lipid oxidation reactions or through the interaction of lipid molecules with the reactive intermediates formed during MR (Whitfield & Mottram, 1992).

**FIGURE 4:**
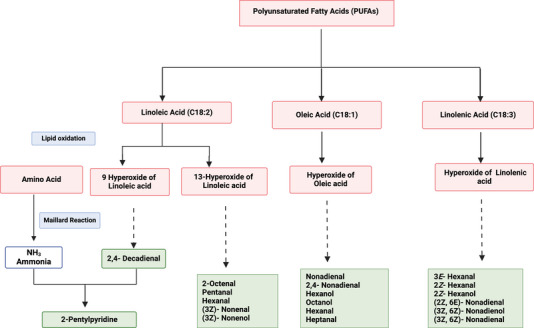
Schematic representation of the proposed mechanism for formation of lipid‐derived VOCs in foods. Light red‐shaded boxes indicate the precursors and intermediates of the reactions. Light green‐shaded boxes represent the volatile compounds formed from the different reactions that have been identified in sweetpotato. Light blue‐shaded boxes indicate the name of the reactions involved. Dashed lines indicate multiple steps in the reaction. Mechanism modified from Vincenti et al. (2019) and Shahidi and Hossain (2022).

Lipid oxidation, a series of complex reactions that produce VOCs important for both desirable and off flavors in crops has been reviewed by numerous authors (Parker, 2015; Shahidi & Hossain, 2022; Shahidi & Oh, 2020; Whitfield & Mottram, 1992). It can occur through autoxidation, photooxidation, and/or enzyme‐catalyzed oxidation (lipoxygenase pathway). Autoxidation is the most common in foods and is accelerated in the presence of light, heat, and metal ions (McClements & Decker, 2007). PUFA and MUFA autooxidation is initiated by the reaction with a free radical, a compound with an unpaired electron (e.g., hydroxyl radical, ∙OH), resulting in peroxide radicals. These hydroperoxides release a hydroxyl radical, forming alkoxyl radicals, which then undergo a cleavage reaction at the beta position of the carbon chain, leading to the formation of various VOCs. For example, linoleic acid can form 9‐ and 13‐hydroperoxides, which degrade into 2,4‐decadienal, 2‐octenal, pentanal, and hexanal (Figure 4; García‐Martínez et al., 2009; Grebenteuch et al., 2021; Yang et al., 2007), while oxidation of oleic acid can lead to formation of octanal, hexanal, and heptanal (Xu et al., 2017). These lipid‐derived oxidative breakdown VOCs have been associated with pleasant flavors in cooked potato, potato chips, cooked rice, cooked beef, corn oil, and wheat bread (Parker, 2015). However, some of the autoxidation products are known to impart off or rancid flavors in deep‐fried sweetpotato crisps (Agarwal et al., 2021).

Lipoxygenases (LOXs) are enzymes that catalyze lipid oxidation. Expression of specific LOX genes leads to the formation of hydroperoxide intermediates, which are further cleaved by other enzymes such as alcohol dehydrogenase and aldehyde lyase to produce various VOCs in most plants (Vincenti et al., 2019). The presence of LOX enzymes in sweetpotato has been reported, but these enzymes have not yet been associated with the formation of aroma compounds. For example, a low expression of *IbLOX1* gene was connected to enhanced antioxidative and storage capability in sweetpotato storage roots (Tang et al., 2019). Although LOX is inactivated by high temperatures (Rodrigo et al., 2007), some free radicals may be generated before inactivation, which could rapidly propagate at high temperatures, generating noticeable quantities of VOCs. Aldehydes, ketones, alkanes, and pyridines (in the presence of an amine) are the major reaction products of PUFA degradation in foods; however, the exact mechanism of formation is not yet characterized in cooked sweetpotato.

At high temperatures, carbonyl compounds from MR can also initiate lipid oxidation to generate additional VOCs (Shahidi & Hossain, 2022), and lipid oxidation products that contain a carbonyl may also participate in the MR. It has been reported that 2‐pentyl pyridine could be formed through the interaction of linoleic acid and MR products in fish and meat products (Henderson & Nawar, 1981). Josephson and Lindsay (1987) found that 2,4‐nonadienal and heptenal, two compounds that impart a strong boiled potato aroma, could be generated through a water‐mediated retro‐aldol condensation reaction between linoleic acid and MR byproducts in cooked potato (Josephson & Lindsay, 1987). Similarly, hexanal, 2,4‐decadienal, and 2‐pentylfuran were identified as the compounds generated in greatest amounts when linoleic acid was degraded in the presence of MR and SD intermediates in a cooked potato model system (Mandin et al., 1999). More complex mechanisms, which involve the reaction of lipids with MR and SD intermediates, are known to be important for VOC formation in model systems. For example, 16 times more phenylacetaldehyde was produced when an oil‐in‐water emulsion containing canola oil, water, glucose, phenylalanine, and a surfactant was heated, compared to an aqueous solution (Troise et al., 2020). This process is suspected to be active in sweetpotato as it contains carbohydrates, lipids, and amino acids (Guo & Mu, 2011). Further research is needed to understand the mechanistic process of the formation of lipid‐derived VOCs and their interaction with other components in cooked sweetpotato.

#### 3.2.5 VOCs formed through thermal release of glycosidically bound terpenes

Sweetpotatoes contain both free (volatile) and glycosidically bound (non‐volatile) terpene compounds (Ohta et al., 1990). The free terpenes are aroma‐active, but the glycosidically bound terpenes only contribute to aroma after hydrolysis from the sugar moiety (Rodriguez et al., 2023). Among six commercial cultivars, glycosidically bound monoterpenes and sesquiterpenes were present in both sweetpotato flesh and peel (Rodriguez et al., 2023). These compounds have been shown to be aroma‐active in cooked sweetpotato. For example, monoterpene esters‐like methyl geranate and monoterpene alcohols such as geraniol and linalool were determined to impart sweet and floral notes through a GC‐O analysis (Wang & Kays, 2000). Sesquiterpenes such as α‐copaene, β‐farnesene, α‐bisabolene, and nerolidol are known for their contribution to earthy, baked potato, woody, and rose‐like aromas, respectively, in a GC‐O study in cooked sweetpotato and model systems (Horvat et al., 1991; Ohta et al., 1990; Shen et al., 2024). β‐elemene, δ‐elemene, and α‐selinene are major aroma components in the essential oils from dried sweetpotato (Marques et al., 2022) and have also been identified in cooked sweetpotato (Shen et al., 2024; Yao et al., 2023). However, their contributions to the cooked sweetpotato aroma are still unknown.

**FIGURE 5:**
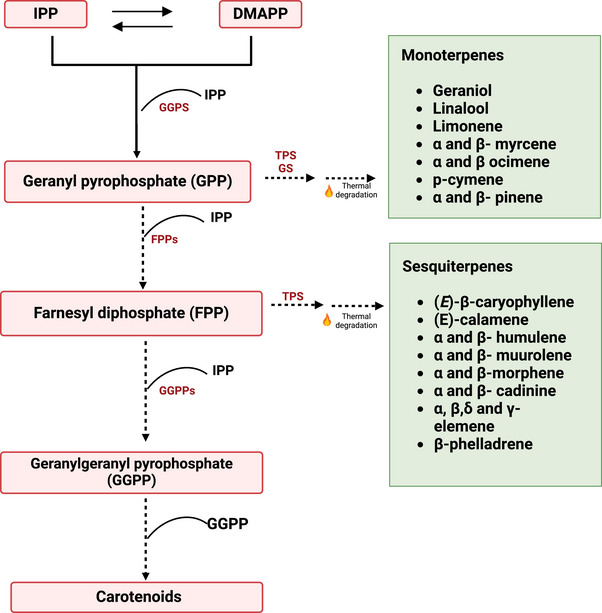
Schematic representation of proposed mechanism for the formation terpene derived VOCs in sweetpotatoes. The figure depicts both the formation of terpenoids in raw sweetpotatoes and their subsequent thermal degradation during cooking. Light red‐shaded boxes indicate the precursors and intermediates of the reactions. Light green‐shaded boxes represent the VOCs formed from the different reactions that have been identified in sweetpotato. Dashed lines indicate multiple steps in the reaction. DMAPP, dimethylallyl pyrophosphate; FPPs, farnesyl pyrophosphate; GGPS, geranylgeranyl pyrophosphate synthase; GS, glucosidases; IPP, isopentenyl diphosphate; TPS, terpene synthase. Figure modified from Rodriguez et al. (2023) and Marques et al. (2022).

The pathway for the synthesis of terpenes shares similar reactions with the pathways for carotenoid biosynthesis in sweetpotato (Kim et al., 2020). It starts with the condensation of dimethylallyl pyrophosphate (DMAPP) with isopentenyl pyrophosphate (IPP) to C5–C25 polyprenyl diphosphate intermediates, a reaction catalyzed by trans/cis‐prenyltransferases (Dudareva et al., 2013). With subsequent addition of IPP, these C5–C25 polyprenyl diphosphate intermediates are subsequently utilized to form the basic skeletons of monoterpenes (C10), sesquiterpenes (C15), diterpenes (C20), and tetraterpenes (C40), including carotenoids, catalyzed by terpene synthase/cyclase enzymes (Chen et al., 2004; Dudareva et al., 2013; Rodriguez et al., 2023; Sharkey et al., 2013). For example, geranyl pyrophosphate undergoes an oxidative degradation reaction catalyzed by monoterpene synthase and cyclases to form D‐limonene, linalool, and geraniol (Rodriguez et al., 2023). Similarly, farnesyl diphosphate can be degraded to a VOC derivative (such as β‐caryophyllene and farnesene) through a series of reactions catalyzed by sesquiterpene synthase and cyclases (Figure 5; Dudareva et al., 2013). Studies in other crops reported high amounts of limonene and linalool as important aroma compounds of oranges and strawberries, respectively (Schwab et al., 2015). Raw sweetpotatoes contain glycosylated terpenes (terpenes bound to sugar molecules) that are non‐volatile, odorless, and act as a storage mechanism (Kays & Wang, 2003). These glycosylated terpenes release volatile terpenes only during hydrolysis or heat treatment, contributing to the aroma profile of the cooked roots. In an effort to understand the contribution of monoterpene alcohols on the aroma of *Sochu, a* popular Japanese sweetpotato drink, Ohta et al., 1990 demonstrated that glycosidically bound monoterpene alcohols could be hydrolyzed by the glycosidic enzymes in steamed sweetpotato. Under acidic conditions (pH 3.8) or elevated temperatures (100°C), geraniol and nerol were converted to linalool and α‐terpineol in *Sochu* (Ohta et al., 1990). Other studies reported that linalool and α‐terpineol were initially absent in raw storage roots but identified in high quantities after acid hydrolysis and baking (Rodriguez et al., 2023; Wang & Kays, 2001). A number of these terpene alcohol products such as linalool, α‐terpineol, citronellol, nerol, and geraniol have been reported in high amounts in recent studies on cooked sweetpotato (Jiang et al., 2023; Tsai et al., 2021; Yao et al., 2023). The exact mechanism involved in terpene degradation during cooking needs to be further elucidated as breeding for varieties that optimize terpene release may enhance the flavor and aroma profiles of sweetpotato.

#### 3.2.6 VOCs formed through carotenoid degradation

**FIGURE 6:**
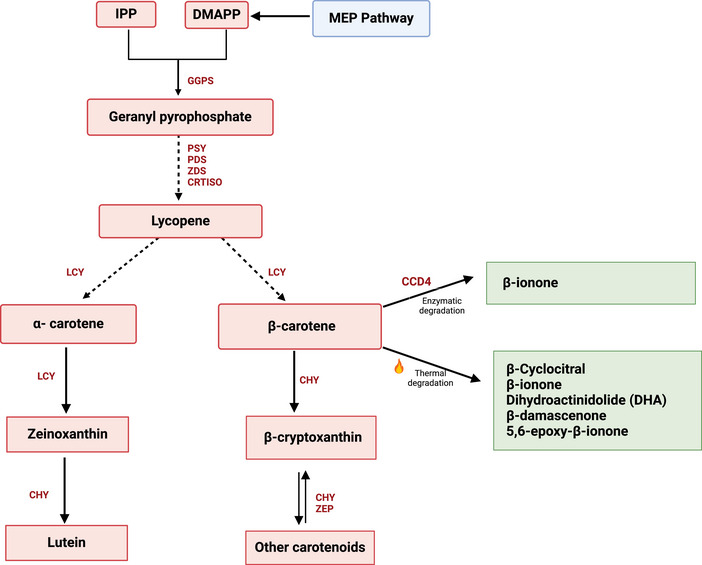
Schematic representation of the proposed mechanism for formation of carotenoid‐derived VOCs in foods. The figure depicts both the formation of carotenoids in raw sweetpotatoes through the and their subsequent thermal degradation during cooking. Red‐shaded boxes indicate the precursors and intermediates of the reactions. Green‐shaded boxes represent the VOCs formed from the different reactions that have been identified in sweetpotato. Yellow‐shaded boxes indicate the name of the reactions involved. Dashed arrows indicate multistep biosynthetic reactions. MEP, methyl‐D‐erythritol phosphate; CHY, carotene hydroxylase; CCD4, carotenoid cleavage dioxygenases 4; CRTISO, carotenoid isomerase; DMAPP, dimethylallyl pyrophosphate; GGPS, geranylgeranyl pyrophosphate synthase; IPP, isopentenyl diphosphate; LCY, lycopene cyclase; PDS, phytoene desaturase; PSY, phytoene synthase; ZDS, zeaxanthin desaturase; ZEP, zeaxanthin epoxidase. Modified from Wu et al. (2018) and Kim et al. (2020).

OFSP can contain 23 different carotenoids that are biosynthesized through a series of reactions between IPP and DMAPP in the methyl‐D‐erythritol phosphate (MEP) pathway, a process that has been elaborately described in many plants (Drapal & Fraser, 2019; Sun et al., 2020), including sweetpotato roots (Firon et al., 2013; Kim et al., 2014; Wu et al., 2018). Along the pathway, carotenoids are subjected to enzyme‐catalyzed oxidative cleavage reactions as well as to non‐enzymatic degradation processes, which produce various carbonyl products called apocarotenoids (Figure 6). These compounds have been identified in dehydrated sweetpotatoes due to oxidation (Bechoff et al., 2010). First, an oxidative cleavage reaction of a carotenoid (catalyzed by a family of carotenoid dioxygenases) leads to the formation of non‐volatile precursors, which further undergoes an acid‐catalyzed conversion to volatile compounds (Bechoff et al., 2010; Tomlins et al., 2012). Achir et al. (2014) reported the primary products from this oxidation reaction of carotenoids at the earlier stages include the 5,6‐epoxide of β‐carotene, while the products at the latter stages are oxidized into volatile lighter carbonyl compounds called apocarotenoids (Achir et al., 2014).

VOCs formed from carotenoid degradation, including β‐ionone, dihydroactinidiolide (DHA), β‐cyclocitral, geranyl acetone, β‐damascenone, and 5,6‐epoxy‐β‐ionone, have been detected in high abundance in cooked sweetpotato (Jiang et al., 2023; Sun et al., 2020; Tsai et al., 2021) and are known to impart violet, woody, sweet‐floral, tea‐like, rose, and fruity aromas in many fruits, vegetables, wines, and teas (Winterhalter & Rouseff, 2002). Of these compounds, β‐ionone was also reported to impart violet aroma in isolated form through a GC‐O analysis (Wang & Kays, 2000). While other VOC intermediates could be generated, β‐ionone and DHA were the first products formed during thermal degradation of β‐carotene in cooked sweetpotato (Aisman et al., 2024). The authors extracted carotenoids from OFSP and heated the extract using an oxidation apparatus while sampling at different time points up to 4 h. They further compared the VOC components formed during the degradation process using GC‐MS and found that the levels of β‐ionone and DHA increased along with decreased total carotene value during heating (Aisman et al., 2024). A similar degradation process has been previously reported in carrot (Syukri et al., 2021). Meanwhile, Kanasawud and Crouzet (1990) showed that 5,6‐epoxy‐β‐ionone, another VOC, could be one of the intermediates formed during the thermal degradation of β‐carotene to β‐ionone in an aqueous medium (Kanasawud & Crouzet, 1990). Other compounds formed from the later stages of thermal β‐carotene degradation include β‐damascenone (tea‐like), β‐cyclocitral (rose), and nerol (fruity) as reported in enzyme kinetic studies in dried sweetpotato chips at low‐temperature conditions (Achir et al., 2014; Bechoff et al., 2010; Tomlins et al., 2012).

Two carotenoid degradation products, (β‐ionone and 1,2,4‐trimethylbenzene) were found to be abundant in OFSP and were strongly positively correlated (R^2 ^= 0.98, *p* = .0001) with the β‐carotene contents of the clones analyzed (Kays & Wang, 2003). Similarly, Yao et al. (2023) reported that the relative contents of β‐ionone and β‐damascenone were highest in dark OFSP and had a positive correlation (*r* > 0.80) with β‐carotene (Yao et al., 2023). Furthermore, multiple authors reported an increase in the relative abundance of some carotenoid‐derived VOCs and a decrease in β‐carotene content after degradation, irrespective of the genotype analyzed (Aisman et al., 2024; Kays & Wang, 2000; Shen et al., 2024; Yao et al., 2023), suggesting that β‐carotene is the main precursor of most of these VOCs. Further studies could provide a detailed mechanism of formation of these important compounds in cooked roots.

Carotenoids may not only produce desirable flavors but can also interact with their metabolites in food matrices to induce off flavors (Sun et al., 2020). For example, Chedea and Jisaka (2013) reported that LOXs can co‐oxidize carotenoids and induce off‐flavor in carotenoid‐rich foods (Chedea & Jisaka, 2013); however, the extent to which this occurs in sweetpotato is not yet known. Since sweetpotatoes are rich in carotenoids and LOXs, it will be worthwhile to explore how these interactions influence off‐flavor development in cooked storage roots. Additional studies could also inform the influence of temperature conditions during cooking in the formation of these important VOCs.

### 3.3 Instrumental analysis of sweetpotato VOCs

Instrumental and sensory analyses have advanced our understanding of what influences aroma and flavor in cooked sweetpotato (Allan et al., 2024; Nakitto et al., 2022; Zhang et al., 2021). A variety of different methods are employed to analyze VOCs from food and fruit samples. This process usually begins with the extraction of compounds, followed by separation via GC and detection by MS, which also enables identification (Horvat et al., 1991; Koehler & Kays, 1991; Tiu et al., 1985).

**FIGURE 7:**
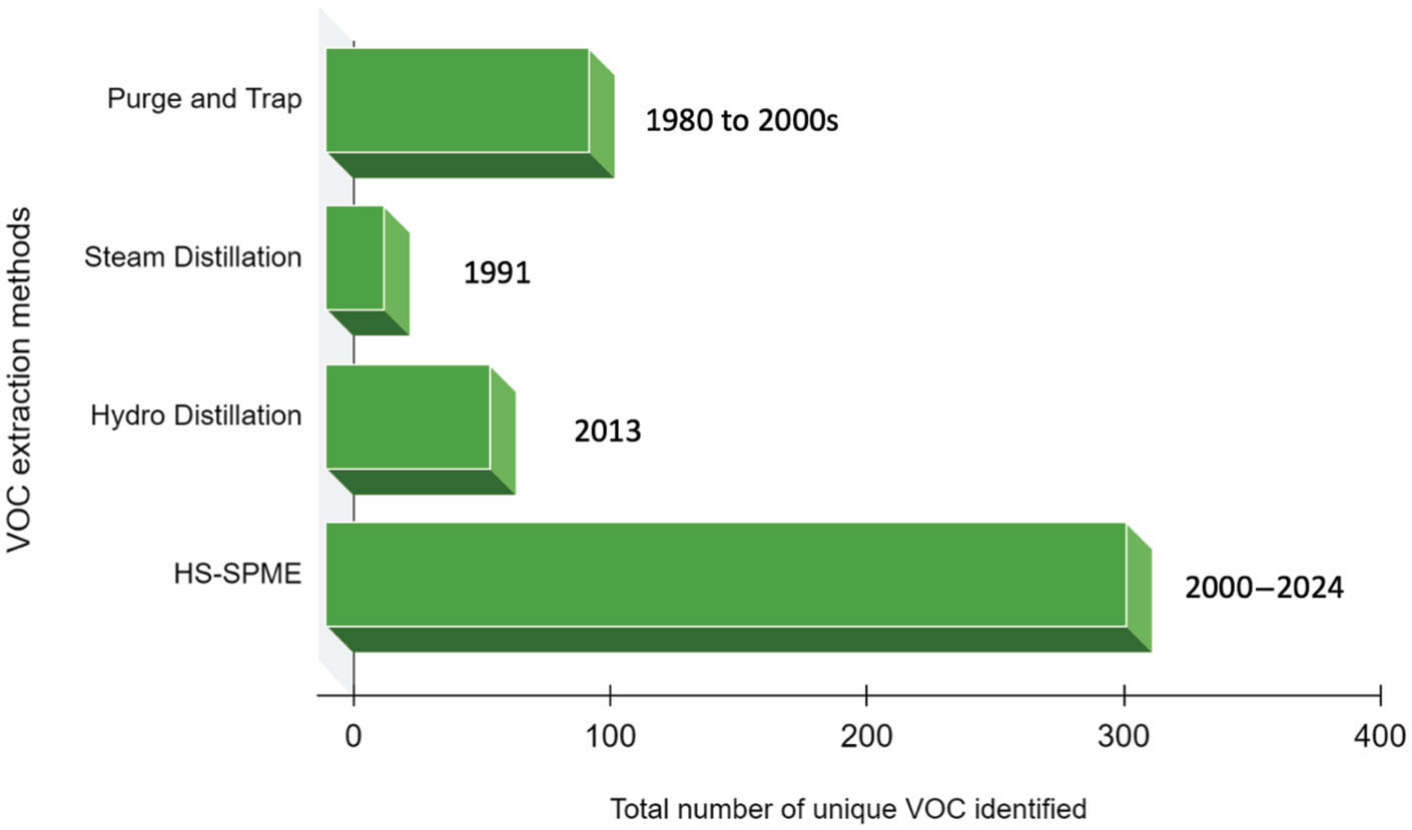
Methods applied in VOC extraction from cooked sweetpotato from 1980 ‐ 2024, including the total number of unique compounds identified using each method. From 1980 to 2000, purge and trap extraction methods were commonly used for VOCs analysis. Only one study in 1991 applied the steam distillation method, which identified 21 compounds, and another study in 2013 applied a hydro‐distillation method, identifying about 75 compounds in boiled sweetpotato. Since the 2000s, there has been a shift in the application of these two methods to the more efficient HS‐SPME method for sweetpotato VOC analysis.

The different extraction methods applied to analyze volatile compounds in sweetpotato include steam distillation (Horvat et al., 1991; Wang & Kays, 2000), purge and trap (Purcell et al., 1980; Tiu et al., 1985), simultaneous distillation and extraction (Sun et al., 1995), and solid‐phase microextraction (SPME; Dumas et al., 2005; Jiang et al., 2023; Nakamura et al., 2013; Shen et al., 2024; Tsai et al., 2021; Yao et al., 2024; Zhang et al., 2021; Table 2). In the purge and trap method, inert gas is used to purge the volatile compounds out of the sample, which are retained in an adsorbent trap. This method is not suitable for quantification (Purcell et al., 1980). The steam distillation and simultaneous distillation and extraction methods are based on a distillation system using water vapor followed by solvent extraction, but it requires the use of large amounts of multiple organic solvents and long extraction times (Zhang & Li, 2010). Headspace SPME (HS‐SPME) offers multiple advantages, compared to other extraction methods including integrating the extraction, concentration, and introduction in one step. HS‐SPME simultaneously results in reduced preparation time and increased sensitivity over other extraction methods (Lancioni et al., 2022). From 1980 to 2000, purge and trap extraction methods were commonly used for VOC analysis. Only one study in 1991 applied the steam distillation method, which identified 21 compounds, and another study in 2013 applied a hydro‐distillation method, identifying about 75 compounds in boiled sweetpotato. Since the 2000s, there has been a shift in the application of these two methods to the more efficient HS‐SPME method for sweetpotato VOC analysis (Figure 7).

**TABLE 3:** Flavor lexicons utilized for descriptive sensory analysis in cooked sweetpotato.

Reference	Aroma descriptors	Flavor descriptors	Number of panelists	Method of cooking	Sweetpotato flesh color
Leighton et al. (2010)	Earthy, sweetpotato, burn	Sweetpotato, yellow vegetables (butternut, carrot, pumpkins), sweet	10	Boiled	Orange, white
Leksrisompong et al. (2012)	Brown sugar, potato, earthy/canned carrot, dried apricot/floral, vanilla	Brown sugar, potato, earthy/canned carrot, vanilla, sour, sweet, bitter, umami, astringent	10	Baked	Orange, white, yellow, purple
Nakitto et al. (2022)	Sweetpotato, caramel, pumpkin, off‐odor	Sweetpotato pumpkin, cooked carrot, floral, sweet, bitter	21	Boiled	Orange, white, yellow, cream
Shen et al. (2024)	Caramel, floral, earthy, sour, burnt, green	–	10	Roasted	Orange white, yellow, purple

HS‐SPME combined with GC‐MS has been demonstrated to be a useful tool in identifying VOCs in sweetpotato (Zhang et al., 2021). In sweetpotato VOCprofiling studies, GC‐MS or GC with a flame‐ionized detector (FID) have been commonly used to quantify the relative abundance of each compound (Koehler & Kays, 1991; Sun et al., 1995; Wang & Kays, 2000). However, these studies have been limited by the concentration of the compounds and the complexity of the sweetpotato matrix, which includes a diverse range of primary and secondary metabolites (Drapal et al., 2019). The development of more efficient identification and detection methods, such as a comprehensive 2‐dimensional gas chromatography (GC × GC) coupled to a time‐of‐flight mass spectrometer (ToFMS), have improved the efficiency of VOC profiling, especially for trace compounds (Mondello et al., 2008). The application of this system in food has been reviewed (Stilo et al., 2021), and its efficiency in untargeted metabolomics was recently demonstrated in cooked sweetpotato (Shen et al., 2024). Using GC×GC‐ToFMS, Shen et al., 2024 identified 120 compounds in roasted sweetpotato of different flesh colors. This accounts for more than an 80% increase in the number of VOCs reported when only GC‐MS was utilized in roasted sweetpotato of different flesh colors (Yao et al., 2023). Therefore, these advancements will aid in the elucidation of key aroma compounds in sweetpotatoes and their source.

### 3.4 Sensory studies of sweetpotato flavor

Sensory evaluation is one of the major tools that can improve our understanding of the contributions of VOCs to perceived flavors and consumer preferences. Such studies could equip breeders with information to guide the development of improved varieties that meet consumer preferences. Among the many sensory analysis methods available, quantitative descriptive analysis (QDA) and consumer acceptance testing have been applied in evaluating the perceptions of different flavor attributes in cooked sweetpotato; and the check‐all‐that‐apply (CATA) technique has been applied in studying regional preferences. In QDA, individuals are trained extensively to identify subtle and objective differences in specific sensory attributes. A sensory lexicon is usually developed to guide the evaluation process. During lexicon development, panelists evaluate samples that represent as much as possible the entire range of products. They generate descriptive terms for the samples, define these terms, establish standardized evaluation procedures, and select references that clarify the terms. These references and samples are then utilized to train panelists and review the list of sensory attributes (Drake & Civille, 2003). The final list of terms utilized for sensory evaluation is selected after a series of evaluations and revisions to guide QDA studies. In contrast, acceptance testing relies on naive consumers who are asked to rate a food product on overall liking and sometimes on their opinions about preferred levels of a range of sensory attributes to identify threshold levels at which they are preferred by consumers. In the CATA method, participants are given a list of attributes and asked to identify all the sensory attributes that are noticeable in a product. It is often used in conjunction with consumer acceptance testing to inform which product characteristics are influencing liking. Each of these methods has its strengths and limitations, and there is ample opportunity for further work in understanding the perception of sweetpotato flavor.

A limited number of studies have applied QDA in cooked sweetpotato. For example, Leighton et al. (2010) established a lexicon with three aroma and four flavor attributes (Table 3) to compare the sensory profiles of different cultivars of OFSP with each other as well as with white‐fleshed sweetpotatoes. They reported that OFSP cultivars displayed flavor characteristics of yellow vegetables (such as butternut squash and pumpkin) when compared with white‐fleshed genotypes (Leighton et al., 2010). Similarly, Leksrisompong et al. (2012) studied the impact of sweetpotato flesh colors on overall sensory perception using a lexicon that included six aroma and 10 flavor attributes. In that study, OFSP cultivars were characterized by earthy/canned carrots, dried apricot/floral flavor and aromas, and brown sugar aroma; purple‐fleshed cultivars were characterized by baked potato flavor and aroma and vanilla flavor, while yellow‐fleshed cultivars were characterized by brown sugar and dried apricot flavors (Leksrisompong et al. 2012). More recently, Nakitto et al. (2022) developed a lexicon that comprised 27 sensory attributes, including five aromas and 10 flavors, which shared common terms with some aroma and flavor attributes identified in earlier studies (Leighton et al., 2010; Leksrisompong et al., 2012). This comprehensive lexicon was used to characterize and differentiate among advanced sweetpotato genotypes in the breeding population that possessed a wide range of sensory attributes (Nakitto et al., 2022). QDA studies using sensory lexicons are not only important for enhancing our understanding of the sensory attributes in cooked sweetpotato but can also help in identifying potential VOCs that predict these attributes. For example, Shen et al. (2024) reported six distinct aromas, which were used to correlate sensory attributes to the potential VOC predictors in different colored sweetpotatoes. Compounds such as guaiacol, 1‐octen‐3‐ol, maltol, acetic acid, octanal, and β‐damascenone were positively correlated with burnt, earthy, caramel, sour, green, and floral aromas, respectively, in roasted sweetpotato (Shen et al., 2024). However, to further utilize this information in breeding and guide the selection of desirable/undesirable flavors, acceptance testing is also needed to understand how the unique sensory attributes influence consumer liking of the crop.

Acceptance testing has been applied in several sweetpotato studies to determine regional and cultural preferences of different genotypes (Kays & Wang, 2003), acceptability of genotypes with different flesh color (Leksrisompong et al., 2012), or cooking methods (Barkley et al., 2017). For example, Ssali et al. (2021) utilized acceptance testing to identify the desirable quality attributes that consumers enjoy in sweetpotato French fries. They reported that attributes such as high dry matter content, crispness, and mealiness were most vital for French fries, and those attributes were lacking in OFSP (Ssali et al., 2021). Similarly, in a study aimed at understanding consumer segmentation of fried sweetpotato, Dery et al. (2021) conducted acceptance testing of 17 sweetpotato cultivars, with 332 consumers representing four regions in Ghana and Nigeria. Consumer preference of fried sweetpotato varied by groups. Some groups preferred cultivars that had characteristics of yam flavor and dry texture; while other groups preferred OFSP with ripe plantain and palm nutty flavor (Dery et al., 2021). In a consumer acceptance study of 12 sweetpotato genotypes of varying flesh color, Leksrisompong et al. (2012) showed that flavor and texture, instead of color, most influenced consumer liking of cooked sweetpotato. Consumers liked sweetpotato cultivars that exhibited a sweet taste, brown sugar, and dried apricot flavor but disliked bitter, umami, and vanilla aromas (Leksrisompong et al., 2012). Furthermore, Tsai et al. (2021) evaluated the effect of roasting on the quality, VOC composition, and consumer liking of sweetpotato using a combination of QDA and acceptance testing (Tsai et al., 2021). They showed that roasting for longer hours (1–2 h) resulted in the highest consumer liking of roasted sweetpotato due to the color, texture, and overall aroma (Tsai et al., 2021). The authors further reported that three VOCs (2‐furanmethanol, furfural, and maltol) were the main sources of the aroma of roasted sweet potatoes, among which 2‐furanmethanol appeared to be related to overall consumer liking (Tsai et al., 2021).

The CATA method has not been applied to evaluate the preferences of specific flavor attributes and VOCs compositions in sweetpotato; however, a few studies have used it to inform the liking of new cultivars that had unique flavor and texture profiles. For example, CATA was used in a consumer acceptability study of four sweet potato cultivars cooked with different methods across multiple cities in Argentina. There were differences in consumer preferences between the cities, which were influenced by flavor and textures of the commonly consumed varieties in each location (Sosa et al., 2024). In addition, a CATA study in Uruguay showed that consumers preferred the traditional purple‐skin OFSP due to their flavor and texture, which drove positive sensory expectations (Lado et al., 2020). While these studies focused on consumer preferences of sweetpotato based on commonly measured attributes like sweetness, texture, and overall flavor, there is limited information on the unique aroma and flavor attributes that influence consumer liking or preference of cooked sweetpotato. Integrating sensory evaluation through both descriptive and acceptance studies can significantly enhance our understanding of key flavor predictors to streamline the target flavor traits to a few compounds and enable more research on their mechanism of formation and genetics. By linking consumer preferences to specific aroma compounds, breeders can better target the selection of sweetpotato varieties that align with consumer needs, ultimately guiding the development of preferred flavor profiles.

### 3.5 Factors influencing the composition of VOCs and flavor attributes in cooked sweetpotato

Numerous factors including genotype, postharvest curing, processing, and cooking methods are known to influence VOC composition and the resultant flavors in cooked sweetpotato; however, only a few of these studies have been reported to date. For example, curing increases synthesis of amylase, which hydrolyzes starch into dextrins and maltose that participate in the MR during cooking—leading to the generation of more VOCs (Wang et al., 1996). Additionally, genotype affects the type of VOCs generated and the perception of flavor. For example, OFSP that are known to be sweet (e.g., Jewel and Centennial) yielded higher levels of MR/caramelization VOCs, while the white‐fleshed genotypes (e.g., GA90‐167) that were perceived to be floral were abundant in terpene‐derived VOCs (Kays & Wang, 2003). Furthermore, white‐fleshed genotypes were abundant in terpene VOCs, and purple‐fleshed genotypes were abundant in smoky and woody‐type aroma VOCs (e.g.,α‐guaiene and guaiacol); OFSP were abundant in VOCs associated with green, grassy, and floral aromas (e.g., α‐terpinene, γ‐terpinene, terpinen‐4‐ol, and β‐ionone); while yellow‐fleshed genotypes were abundant in green and earthy VOCs (e.g., 4‐isopropylbenzyl alcohol andα‐patchoulene). Both studies reported that the starch and sugar content significantly influenced the differences in VOC compositions, as well as the flavor attributes observed in different genotypes (Shen et al., 2024; Kays & Wang, 2003).

Four studies evaluated the impact of cooking methods on VOC composition in sweetpotato. In OFSP, there was a 54.3% and 6.4% reduction of total VOCs in boiled and microwaved sweetpotatoes, respectively, when compared with conventional baking (Wang & Kays, 2001). Yao et al. (2023) confirmed this result and showed that baked OFSP generated the highest number of VOCs (*n* = 30), compared to steamed (*n* = 27), boiled (*n* = 24), and raw (*n* = 20). However, this pattern differed for purple‐ and yellow‐fleshed genotypes. In purple‐fleshed genotypes, steaming resulted in the highest number of VOCs (*n *= 35) followed by boiled (*n* = 31), raw (*n* = 29), and baked (*n* = 24) sweetpotatoes (Jiang et al., 2023); and yellow‐fleshed genotypes had the highest number of VOCs after steaming (*n* = 53), followed by baked (*n* = 52), boiled (*n* = 48), and raw (*n* = 35) sweetpotatoes (Zhang et al., 2023). Taken together, these studies have shown that cooking method can impact VOC composition in sweetpotato; but it is not known how these changes in VOC composition translate to the perception of different sensory attributes.

## 4 A ROADMAP OF OPPORTUNITIES FOR FLAVOR BREEDING IN SWEETPOTATO—*the role of genetics*

Currently, conventional breeding and new genetic tools such as quantitative trait loci (QTL) mapping, genomic selection, and genome‐wide association studies (GWAS) are being applied to identify important agronomic and quality traits in sweetpotato (Cervantes‐Flores et al., 2011; Gemenet, Da Silva Pereira, et al., 2020; Gemenet, Kitavi, et al., 2020; Pereira et al., 2020; Yada et al., 2017). However, to our knowledge, no study has been conducted to identify potential genes associated with the formation of VOC precursors in cooked sweetpotato. While current literature primarily focuses on the identification of VOCs and their relationship to flavor and aroma, recognizing the key constituents influencing sweetpotato flavor opens avenues to uncover the genetic mechanisms, such as enzymes and specific metabolites, that control their accumulation. This knowledge can be combined with further metabolomic studies, linkage mapping studies, and metabolome‐based GWAS to understand the extent of natural variation in the genetic control of flavor genes in sweetpotato and help plant breeders to develop new varieties with exceptional flavor profiles. Similar studies have been conducted in tomato (Zhu et al., 2018) and informed selection in blueberries (Colantonio et al., 2022).

The recent release of a fully sequenced sweetpotato reference genome (https://sweetpotato.uga.edu/; Wu et al., 2018; Yencho et al., 2025) offers a framework for identifying QTLs and development of molecular markers that can enable breeders to select for consumer‐preferred flavors. Additionally, flavor‐related metabolites identified in metabolomic studies can be utilized to profile many sweetpotato breeding lines to enable GWAS, focused on identifying the genes/loci contributing to flavor and developing molecular genetic tools for marker‐assisted breeding (MAB). The use of MAB information from this process can speed up the genetic enrichment for flavor‐associated traits (Yan et al., 2022). However, MAB alone cannot address the complexity of flavor perception. Unlike agronomic and yield‐related traits, most flavor traits in foods are known to have large variability, low heritability, and are subjected to complex interaction effect as shown in genetic prediction studies in bread (Longin et al., 2020), apples (Kouassi et al., 2009), and sweet pepper (Eggink et al., 2012). Genomic selection, which utilizes genome‐wide DNA markers for complex traits and across multiple environments could be used to predict breeding values for flavor‐related traits without the need for phenotyping during the later stages of breeding.

Moreso, a multi‐omics approach in functional genomics studies could be used to determine the genes and the gene networks controlling the expression of target VOC traits (Yan et al., 2022). These studies have been applied in making flavor breeding decisions for other crops, including strawberries (Fan et al., 2022), watermelon (Gong et al., 2023), peach (Cao et al., 2024), and other vegetables (Zhu et al., 2019). Furthermore, cutting‐edge genetic tools such as genetic engineering and gene editing, which have recently been applied to enhance VOC and flavor profiles in various horticultural crops (Kaur et al., 2023), hold significant potential for developing sweetpotatoes with improved flavor profiles. These advancements could fast track flavor improvement, resulting in increased consumer satisfaction and greater market demand, reinforcing the importance of integrating genetic insights into consumer preferred sweetpotato breeding efforts.

## 5 CONCLUSION

This review describes the body of work done to characterize the flavor components of cooked sweetpotato. Our review of 22 articles dedicated to sweetpotato VOC identification shows that over 400 VOCs have been identified in cooked sweetpotato, with 76 of them reported to be aroma‐active. While there is notable overlap among VOCs found in major studies, there are discrepancies on the relative abundances and classifications of these compounds identified in cooked sweetpotato. Multiple factors may be responsible for these differences, including the method of cooking, genotypes analyzed, and quantification method. Additionally, very few studies have used sensory evaluation to connect VOC profiles to the aroma or flavor profiles of cooked sweetpotato. There are opportunities to further identify the key predictors of unique aroma and flavor profiles in cooked sweetpotato and their mode of interactions by better integration of analytical chemistry and sensory science. Some of these future research efforts should focus on bringing together a non‐targeted and comprehensive list of all volatile compounds present in commercially available cultivars and their relative concentration. A deeper understanding of how these compounds influence the human sensory perception of sweetpotato flavor will also be needed to ascertain which VOCs predict desirable or undesirable aromas and flavors and how these perceptions are influenced by multi‐modal interactions. Understanding how other factors play a role in controlling VOCs and flavor in sweetpotato will enable breeders to select and develop sweetpotato varieties with specific, consumer‐preferred flavor profiles. Moreso, information about the mechanisms of formation, precursor molecules, potential genes involved, and factors influencing the production of key compounds will go a long way in breeding sweetpotato for flavor. By focusing on cultivars that contain desirable volatile compounds and essential flavor precursors, breeders can create sweetpotatoes with enhanced flavors that appeal to different markets and consumer segments.

## AUTHOR CONTRIBUTIONS

**Modesta Abugu**: Conceptualization; data curation; formal analysis; visualization; writing—original draft; methodology; writing—review and editing. **Matthew Allan**: Writing—review and editing; resources. **Suzanne Johanningsmeier**: Supervision, project administration; writing—review and editing; conceptualization. **Massimo Iorizzo**: Writing—review and editing; supervision; methodology; conceptualization. **G. Craig Yencho**: Supervision; project administration; writing—review and editing; methodology; conceptualization; funding acquisition; resources.

## CONFLICT OF INTEREST STATEMENT

The authors declare no conflicts of interest.

## Supporting information



Supporting Information

Supporting Information
