# Supplementary material for: Comprehensive review of sweetpotato flavor compounds: Opportunities for developing consumer‐preferred varieties
Source: Compr Rev Food Sci Food Saf. 2025 Apr 24;24(3):e70172. doi: 10.1111/1541-4337.70172 (PMC12019920; doi:10.1111/1541-4337.70172)
Supplement: Supplementary file 1 — Supporting Information [file CRF3-24-e70172-s002.docx]

**S1 Material 1. Search strategy, included, and excluded articles full list**

**All information provided below is based on final searches performed on July 15^th^, 2024**

**Table S1:** The Boolean search strategy to identify and collect studies that examined the volatile organic acid compositions in cooked sweetpotatoes

|  | **WEB OF SCIENCE** |  | **CAB ABSTRACTS** |  | **FSTA** |  |
| --- | --- | --- | --- | --- | --- | --- |
| 1 | (TS=(("Sweet potato" OR "ipomoea batatas" OR "North American sweetpotato" OR sweetpotato))) AND TS=((Flavor OR flavour OR volatile OR "Organic compounds" OR sugars OR"Flavor chemistry" OR "flavour chemistry" OR "Flavour profiles" OR "flavor profiles" OR "Volatile compounds" OR "Organoleptic compounds" OR "Sweet taste" OR sweetness OR "Organic Acids")) | 870 | TI ( "Sweet potato" OR "ipomoea batatas" OR "North American sweetpotato" OR sweetpotato ) AND TI ( Flavor OR flavour OR volatile OR "Organic compounds" OR sugars OR"Flavor chemistry" OR "flavour chemistry" OR "Flavour profiles" OR "flavor profiles" OR "Volatile compounds" OR "Organoleptic compounds" OR "Sweet taste" OR sweetness OR "Organic Acids" ) | 110 | TI ( "Sweet potato" OR "ipomoea batatas" OR "North American sweetpotato" OR sweetpotato ) AND TI ( Flavor OR flavour OR volatile OR "Organic compounds" OR sugars OR"Flavor chemistry" OR "flavour chemistry" OR "Flavour profiles" OR "flavor profiles" OR "Volatile compounds" OR "Organoleptic compounds" OR "Sweet taste" OR sweetness OR "Organic Acids" ) | 80 |
|  | AND |  |  |  |  |  |
| 2 | (TI=(("Sweet potato" OR "ipomoea batatas" OR "North American sweetpotato" OR sweetpotato))) AND TI=((Flavor OR flavour OR volatile OR "Organic compounds" OR sugars OR"Flavor chemistry" OR "flavour chemistry" OR "Flavour profiles" OR "flavor profiles" OR "Volatile compounds" OR "Organoleptic compounds" OR "Sweet taste" OR sweetness OR "Organic Acids")) | 107 | AB ( "Sweet potato" OR "ipomoea batatas" OR "North American sweetpotato" OR sweetpotato ) AND AB ( Flavor OR flavour OR volatile OR "Organic compounds" OR sugars OR"Flavor chemistry" OR "flavour chemistry" OR "Flavour profiles" OR "flavor profiles" OR "Volatile compounds" OR "Organoleptic compounds" OR "Sweet taste" OR sweetness OR "Organic Acids" ) | 1119 | AB ( "Sweet potato" OR "ipomoea batatas" OR "North American sweetpotato" OR sweetpotato ) AND AB ( Flavor OR flavour OR volatile OR "Organic compounds" OR sugars OR"Flavor chemistry" OR "flavour chemistry" OR "Flavour profiles" OR "flavor profiles" OR "Volatile compounds" OR "Organoleptic compounds" OR "Sweet taste" OR sweetness OR "Organic Acids" ) | 813 |
|  | AND |  |  |  |  |  |
| 3 | (AB=(("Sweet potato" OR "ipomoea batatas" OR "North American sweetpotato" OR sweetpotato))) AND AB=((Flavor OR flavour OR volatile OR "Organic compounds" OR sugars OR"Flavor chemistry" OR "flavour chemistry" OR "Flavour profiles" OR "flavor profiles" OR "Volatile compounds" OR "Organoleptic compounds" OR "Sweet taste" OR sweetness OR "Organic Acids")) | 597 | SU ( "Sweet potato" OR "ipomoea batatas" OR "North American sweetpotato" OR sweetpotato ) AND SU ( Flavor OR flavour OR volatile OR "Organic compounds" OR sugars OR"Flavor chemistry" OR "flavour chemistry" OR "Flavour profiles" OR "flavor profiles" OR "Volatile compounds" OR "Organoleptic compounds" OR "Sweet taste" OR sweetness OR "Organic Acids" ) | 772 | SU ( "Sweet potato" OR "ipomoea batatas" OR "North American sweetpotato" OR sweetpotato ) AND SU ( Flavor OR flavour OR volatile OR "Organic compounds" OR sugars OR"Flavor chemistry" OR "flavour chemistry" OR "Flavour profiles" OR "flavor profiles" OR "Volatile compounds" OR "Organoleptic compounds" OR "Sweet taste" OR sweetness OR "Organic Acids" ) | 1439 |
|  | AND |  |  |  |  |  |
|  | 1 AND 2 AND 3 | 74 | 1 AND 2 AND 3 | 70 | 1 AND 2 AND 3 | 63 |

Additional keywords manually keyed into google scholar include “baked sweetpotato”, “roasted sweetpotato”, “boiled sweetpotato”, “gas chromatography”, “mass spectrometry” or “GC-MS”. Outputs from this search were the same with those generated from the database search.

Results of articles from the above process of the search strategy appear in the following chart.

VOCs composition (N=22)

Sensory evaluation (N=8)

Suggested VOCs mechanisms of formation* (N=37)

Records excluded based on full article review (N=102)

Studies included in review (N=67)

5,739 articles excluded based on title, abstract, or duplication

Total full articles reviewed (N=169)

Total Records (N= 5,907)

Web of Science (N=1,574)

CABAbstracts (N=2,001)

FSTA (N=2,332)

**Fig 1.** Studies screened and selected for inclusion in the review of volatile compositions of cooked sweetpotato

Notes: * These articles are supporting articles used to describe the mechanism of formation of sweetpotato VOCs as reported in sweetpotato model systems and in other crops

Search process started on August 28^th^, 2021, and finished on July 15^th^, 2024. Based on the review of the full text, 102 articles were excluded, and the explanation for exclusion is described in this appendix.

1. **Included Articles List Specific to VOCs Composition in Cooked Sweetpotato (N=22)**
2. Purcell et al., 1980
3. Tiu, et al., 1985
4. Horvat et al., 1991
5. Sun et al., 1993
6. Sun et al., 1994
7. Sun et al., 1995
8. Wang et al., 1998
9. Kays and Wang 2000a
10. Wang and Kays 2000b
11. Wang and Kays 2001
12. Wang and Kays2003
13. Dumas et al 2005
14. Nakamura et al., 2013
15. Hou et al., 2020
16. Ravi et al, 2022
17. Tsai et al., 2021
18. Zhang et al, 2021
19. Yao et al., 2023a
20. Jiang et al., 2023
21. Yao et al., 2023b
22. Zhang et al., 2023
23. Shen et al., 2024

# **Additional Articles Used to Discuss the Mechanism of Formation of Sweetpotato VOCs in both sweetpotato and other crops (N=37)**

1. Achir et al. (2014)
2. Agarwal et al. (2021)
3. Aisman et al. (2024)
4. Allan et al., (2024)
5. Allan et al., (2023)
6. Bechoff et al. (2010)
7. Bordiga et al. (2019)
8. Chedea et al. (2013)
9. Chen et al. (2004)
10. Chu et al. (2008)
11. Dudareva et al. (2013)
12. Feng et al. (2022)
13. Fu et al. (2020)
14. García-Martínez et al. (2009)
15. Gemenet et al. (2020)
16. Grebenteuch (2021)
17. Habinshuti et al. (2021)
18. Hagenimana et al. (1994)
19. Hemmler et al. (2017)
20. Hemmler et al. (2018)
21. Henderson et al. (1981)
22. Hidalgo et al. (2013)
23. Hofmann et al. (2000)
24. Kanzler et al. (2017)
25. Kanasawud et al. (1990)
26. Liu et al. (2022)
27. Mandin et al. (1999)
28. Martins et al. (2000)
29. Mcclements et al. (2008)
30. Parket (2015)
31. Qui et al. (2020)
32. Rodriguez et al. (2023)
33. Schwab et al. (2015)
34. Shahidi et al. (2022)
35. Sun et al. (2020)
36. Tomlins et al. (2012)
37. Troise et al., (2020)

# **Included Articles List Specific to Sensory Analysis, Lexicon Development and Consumer Preferences of Cooked Sweetpotato (N=7)**

1. Barkley et al. (2017)
2. Dery et al. (2021)
3. Leighton et al. (2010)
4. Leksrisompong et al. (2012)
5. Nakitto et al. (2022)
6. Shen et al. (2024)
7. Sosa et al. (2023)
8. Lado et al. (2020)

# **Excluded Articles List (N=102)**

1. Adu-Kwarteng et al. (2014)
   1. Not relevant, measures the effect of harvest and storage time on sugar content in sweetpotato
2. Ayala et al (2020)
   1. not relevant, measures volatile release of sweetpotato whitefly
3. Baafi et al. (2017)
   1. Not relevant. Genetic control of dry matter, starch and sugar content in sweetpotato
4. Baba et al. (1987)
   1. Not relevant. Development of snack foods produced from sweet potatoes. V. Changes in sugar and starch contents during storage of new type sweetpotato
5. Cassily (1971)
   1. Not relevant. Isolates the precursors of off-flavour in oxidized sweet potato flakes
6. Chapman (1989)
   1. Not relevant. Determination of nonvolatile acids and sugars from fruits and sweet potato extracts by capillary GLC and GLC/MS
7. Cheng et al. (2014)
   1. Not related. Comparative study of influence of reducing sugar content and polyphenol oxidase activity of raw sweet potato on the color of sweet potato granules
8. Chun et al. (2003)
   1. Study on quality improvement of sugar curing sweet potato
9. Clark (1989)
   1. Not related. Influence of volatiles from healthy and decaying sweet potato storage roots on sclerotial germination and hyphal growth of Sclerotium rolfsii
10. Cui et al., (2010)
    1. Not relevant. Changes in volatile compounds of sweet potato tips during fermentation
11. Dini et al. (2006)
    1. Not related. Seven new aminoacyl sugars in Ipomoea batatas
12. Hernandez-Carrion et al. (2011)
    1. Not relevant . Sugars and alcohol insoluble solids assessment for sweet potato cultivars recommended for Puerto Rico
13. Hiti mana et al. (2000)
    1. Not relevant. Host plant volatiles to sweet potato butterfly
14. Hong-Jun et al. (2014)
    1. Not relevant. Volatile flavor compounds in regular and purple sweet potato yogurt
15. Hu et al (2010)
    1. Not related. Insecticidal activity of some reducing sugars against the sweet potato whitefly, Bemisia tabaci, Biotype B
16. Hua et al. (2021)
    1. Not related. Functional characteristic analysis of three odorant-binding proteins from the sweet potato weevil (Cylas formicarius) in the perception of sex pheromones and host plant volatiles
17. Hua et al (2021)
    1. Not related. Three chemosensory proteins from the sweet potato weevil, Cylas formicarius, are involved in the perception of host plant volatiles
18. Huang et al (1999)
    1. Not relevant. Changes in invertase activities and reducing sugar content in sweetpotato stored at different temperatures
19. Inukai et al. (2001)
    1. Not relevant .Growth conditions affecting palatability, especially sweetness of sweet potato
20. Januario et al. (2017)
    1. Not related. Physicochemical and texture evaluation, probiotic viability and acceptance of probiotic yoghurt flavored with organic beet with carrot, cassava, sweet potato
21. Jia et al. (2017)
    1. Not related. EAG and olfactory responses of Cylas formicarius (Coleoptera: Curculionidae) to volatiles from plants of different sweetpotato cultivars
22. Jian-lan et al. (2016)
    1. Not relevant. Comparing the volatile components of sweet potato steamed bun and ordinary steamed bun
23. Jang et al. (2018)
    1. Not relevant. Flavor And Stability Improvement Of Purple Sweet Potato - Peanut Compound Beverage
24. Jiamin et al. (2021)
    1. Not relevant. Development of low-sugar compound grain powder with purple sweet potato
25. Rhim, Jong-Whan (2002)
    1. Not relevant. Effects of organic acids on the extraction kinetics of purple-fleshed sweet potato pigment
26. Jeong-Seob (2010)
    1. Not relevant. Effects of sweet potato cultivars and koji types on general properties and volatile flavor compounds in sweet potato soju
27. Kadowaki et al. (2001)
    1. Not relevant. Effects of exogenous injection of different sugars on leaf photosynthesis, dry matter production and adenosine 5 '-diphosphate glucose pyrophosphorylase (AGPase) activity in sweet potato, Ipomoea batatas (Lam.)
28. Kays and Wang (1998)
    1. Not relevant. Development of alternative flavour types of sweetpotato as a means of expanding consumption
29. Ke et al. (2012)
    1. Not relevant. Determination of soluble sugars in sweet potato by HPLC and its changes during processing
30. Kitayama et al. (2020)
    1. Not relevant. Prediction of starch, moisture, and sugar in sweetpotato by near infrared transmittance
31. Koehler and Kays (1991)
    1. Not relevant. Sweet-potato flavor - quantitative and qualitative assessment of optimum sweetness
32. Kohyama and Nishinari et al (1991)
    1. Not relevant. Effect of soluble sugars on gelatinization and retrogradation of sweet potato starch
33. Korada et al. (2013)
    1. Not related. Plant volatile organic compounds as chemical markers to identify resistance in sweet potato against weevil Cylas formicarius
34. Korada et al. (2010)
    1. Not related. Differential volatile emission from sweet potato plant: mechanism of resistance in sweet potato for weevil Cylas formicarius (Fab.)
35. Lebot (2017)
    1. Changes in anthocyanins and volatile components of purple sweet potato fermented alcoholic beverage during aging
36. Lee et al. (2018)
    1. Not relevant. Analyzes flavor of aged spirits made from sweet potato
37. Lewthwaite et al (1997)
    1. Not relevant. Free sugar composition of sweetpotato cultivars after storage
38. Li et al. (2017)
    1. Not relevant. Rapid determination of moisture and reducing sugar in sweet potato by near-infrared spectroscopy coupled with chemometrics
39. Li et al. (2017)
    1. Not relevant. Changes in anthocyanins and volatile components of purple sweet potato fermented alcoholic beverage during aging
40. Liu et al. (2011)
    1. Not relevant. Physiological basis of improving soluble sugar content in sweetpotato for table use by humic acid application
41. Liu et al. (2012)
    1. Not relevant. Effect of potassium on the activities of enzymes related to sugar metabolism of edible sweet potato
42. Li et al. (2014)
    1. Not relevant. Study on technology of low sugar, soft and additive free sweet potato
43. Liu et al. (2020)
    1. Not related. Relationship between sugar content in sweet potato and vibration acoustic signal eigenvalues
44. Li et al (2020)
    1. Unrelated. Identification of Rhizospheric Actinomycete Streptomyces lavendulae SPS-33 and the Inhibitory Effect of its Volatile Organic Compounds against Ceratocystis fimbriata in Postharvest Sweet Potato
45. Martin and Rodriguez-Sosa (1985)
    1. Not relevant. Preference for color, sweetness, and mouthfeel of sweet potato in Puerto Rico
46. Martin et al. (1985)
    1. Not relevant. Sugars and starches in a non-sweet sweet potato compared to those of conventional cultivars
47. Maeo et al. (2001)
    1. Not relevant. Sugar-responsible elements in the promoter of a gene for beta-amylase of sweet potato
48. McDonald et al. (1970)
    1. Not relevant. Extraction and gas liquid chromatography of sweet potato sugars and inositol
49. Mateus et al. (2017)
    1. Not relevant, discussis antioxidant activity and sugar content of sweet potato peels
50. Mensah et al. (2016)
    1. Not relevant. Thermal stability of β-amylase activity and sugar profile of sweet potato varieties during processing
51. Meents et al. (2019)
    1. Not related. Volatile DMNT systemically induces jasmonate-independent direct anti-herbivore defense in leaves of sweet potato (Ipomoea batatas) plants
52. Ming- yu (2016)
    1. Not relevant. Development and flavor substances analysis of purple sweet potato and bitter buckwheat combined vinegar beverage
53. Mirzaei et al. (2021)
    1. Not related. Optimization of consumption levels of sweet potato puree, inulin and date liquid sugar
54. Mortley et al. (2008)
    1. Not related. Influence of microgravity environment on root growth, soluble sugars, and starch concentration of sweetpotato stem cuttings
55. Motsa et al (2015)
    1. Not relevant. Influence of agro-ecological production areas on antioxidant activity, reducing sugar content, and selected phytonutrients of orange-fleshed sweet potato cultivars
56. Muhanna et al. (2004)
    1. Not related. The role of root sugar content on the susceptibility of sweetpotato cultivars to soft rot
57. Myoung et al. (2015)
    1. Not relevant. Discusses flavors of the Soju produced from Korean sweetpotato
58. Nagata and Saitou (2009)
    1. Not related. Regulation of Expression of D3-type Cyclins and ADP-Glucose Pyrophosphorylase Genes by Sugar, Cytokinin and ABA in Sweet Potato
59. Nakamura et al. (2020)
    1. Not relevant. Discusses carbohydrates associated with sweetness of cooked sweetpotato
60. Naidoo et al (2021)
    1. Not related. Combining ability, heterosis and heritability of sweetpotato root protein, beta-carotene, sugars and mineral composition
61. Ohto et al. (1995)
    1. Unrelated. Involvement of ca2+ signaling in the sugar-inducible expression of genes-coding for sporamin and beta-amylase of sweet-potato
62. Owusu-Mensah et al. (2016)
    1. Unrelated. Cooking treatment effects on sugar profile and sweetness of eleven-released sweet potato varieties
63. Patil et al. 92006)
    1. unrelated. Effect of potassium nutrition on dry matter accumulation, sugars, starch and nutrient concentration in sweet potato
64. Pereira et al (2017)
    1. Unrelated. Enzymatic conversion of sweet potato granular starch into fermentable sugars: Feasibility of sweet potato peel as alternative substrate for alpha-amylase production
65. Picha 1986
    1. Not relevant. Influence of storage duration and temperature on sweet potato sugar content and chip color
66. Prandhan et al. (2015)
    1. Not relevant. High starch, low sugar yielding genotypes of sweet potato and their micropropagation
67. Rodriguez et al. (2001)
    1. Unrelated. Dynamic of fermentation of sugar cane and sweet potato mixtures
68. Sacchetti et al (2015)
    1. Not relevant. Volatile organic compounds emitted by bottlebrush species affect the behaviour of the sweet potato whitefly
69. Salelign and Duraisamy (2021)
    1. Not relevant. Sugar and ethanol production potential of sweet potato (Ipomoea batatas) as an alternative energy feedstock: processing and physicochemical characterizations
70. Samantaray and Korada (2016)
    1. Not relevant. Electrophysiological and behavioural responses of sweetpotato weevil, Cylas formicarius to green leaf volatiles and terpenoids
71. Schweinberger et al. (2019)
    1. Not relevant. A simple equation for total reducing sugars (trs) estimation on sweet potato and ethanol yield potential
72. Stroparo et al. (2019)
    1. Not relevant. Evaluation of Sweet Potato Cultivars to the Formation of Sugars with Potential for the Production of Ethanol
73. Sawai et al. (2004)
    1. Not relevant. A comparison of the hydrolysis of sweet potato starch with beta-amylase and infrared radiation allows prediction of reducing sugar production
74. Shen et al. (2017)
    1. Not relevant. Analyzes soluble sugar and taste components in sweetpotato
75. Shu-min et al. (2016)
    1. Not relevant. Study on process of sugar permeation by microwave vacuum technology in preserved fruit making of low sugar purple sweet potato
76. Sun et al. (2010)
    1. Not relevant. Comparison of the effect of sugars on the viscoelastic properties of sweet potato starch pastes
77. Sun et al. (2008)
    1. Not relevant. Flow behavior of sweet potato starch in mixed sugar systems
78. Sukhveer et al. 2017
    1. Not relevant. Formulation and process optimization of phalahari muffin produced from sugar, butter and sweet potato flour
79. Solihin et al. (2018)
    1. Not relevant. Discriminating Land Characteristics of Yield and Total Sugar Content Classes of Cilembu Sweet Potato
80. Son et al. (2019)
    1. Unrelated. Root carbohydrates, organic acids, and phenolic chemistry in relation to sweetpotato weevil resistance
81. Takahata et al. (1992)
    1. Not relevant. Varietal diversity of free sugar composition in storage root of sweet potato
82. Takizawa et al. (2019)
    1. Not relevant. Sweet potato shochu, method for producing sweet potato shochu, and sweet potato shochu flavor improving method
83. Tang et al. (2013)
    1. Not relevant. Prediction of starch and sugar contents in sweet potato root by near-infrared spectroscopy (NIRS)
84. Tisarum et al. (2020)
    1. Unrelated. Foliar application of glycinebetaine regulates soluble sugars and modulates physiological adaptations in sweet potato (Ipomoea batatas) under water deficit
85. Truong et al. (1998)
    1. Not relevant. Simple sugars, oligosaccharides, and starch concentrations in raw and cooked sweet potato
86. Veeraragavathatham et al. (2006)
    1. Not relevant. Sweet potato in Indian cusine: Use of varieties with the least sweetness
87. Wang and Kays 2002
    1. Not relevant. Sweetpotato volatile chemistry in relation to sweetpotato weevil behavior
88. Wang and Kays (2005)
    1. Not relevant. Chemical and geographical assessment of the sweetness of the cultivated sweetpotato clones of the world
89. Wang et al. (2016)
    1. Unrelated. Compares root characteristics and sugar components in sweetpotato root and leaf
90. Xin et al. (2017)
    1. Not relevant. Characterization of root morphology and root-derived low molecular weight organic acids in two sweet potato cultivars exposed to cadmium
91. Xio-ping et al. (2016)
    1. Not relevant. Formulation optimization of low sugar purple sweet potato crisp biscuit
92. Xu et al. (2021)
    1. Not relevant. Discusses antibiotic effects of volatiles produced by Bacillus tequilensis in postharvest sweet potato
93. Wenjuan et al. (2016)
    1. Not relevant. Effects of deodorization on the physicochemical index and volatile compounds of purple sweet potato anthocyanins (PSPAs)
94. Ying et al. (2015)
    1. Not relevant. Effects of cooking methods on starch and sugar composition of sweetpotato storage roots
95. Yang et al. (2017)
    1. Not relevant. Effect of processing methods on sugar, aroma and sweetness of sweetpotato
96. Yavuzer et al. (2020)
    1. Not relevant. Impact of icing with potato, sweet potato, sugar beet, and red beet peel extract on the sensory, chemical, and microbiological changes of rainbow trout fillets
97. Yooyongwech et al. (2016)
    1. Not relevant. Discusses Arbuscular mycorrhizal fungi activity in sweetpotato
98. Yooyongwech et al. (2017)
    1. Not relevant. Water-Deficit Tolerance in Sweet Potato Ipomoea batatas (L.) Lam. by Foliar Application of Paclobutrazol: Role of Soluble Sugar and Free Proline
99. Yu et al. (2017)
    1. Not relevant. Analyzes volatile aroma components in sweet potato vinegar
100. Yue et al. (2014)
     1. Not relevant. The technology research of purple sweet potato flavor walnuts
101. Yu-qing 2017
     1. Not relevant. The formula of flavor sweet potato powder
102. Zhu et al. (2022)
     1. Not relevant. Nutritional composition, antioxidant activity, volatile compounds, and stability properties of sweet potato residues fermented with selected lactic acid bacteria and bifidobacteria

# **References**

Abbas, F., Zhou, Y., O’Neill Rothenberg, D., Alam, I., Ke, Y., & Wang, H.-C. (2023). Aroma Components in Horticultural Crops: Chemical Diversity and Usage of Metabolic Engineering for Industrial Applications. *Plants*, *12*(9), 1748. <https://doi.org/10.3390/plants12091748>

Achir, N., Pénicaud, C., Bechoff, A., Boulanger, R., Dornier, M., & Dhuique-Mayer, C. (2014). Use of Multi-response Modelling to Investigate Mechanisms of β-Carotene Degradation in Dried, Orange-Fleshed Sweet Potato During Storage: From Carotenoids to Aroma Compounds. *Food and Bioprocess Technology*, *7*(6), 1656–1669. <https://doi.org/10.1007/s11947-013-1229-y>

Adu-Kwarteng, E., Sakyi-Dawson, E. O., Ayernor, G. S., Truong, V. D., Shih, F. F., & Daigle, K. (2014). Variability of sugars in staple-type sweet potato (Ipomoea batatas) cultivars: the effects of harvest time and storage. *International Journal of Food Properties*, *17*(2), 410-420.

Adu-Kwarteng, E., Sakyi-Dawson, E. O., Ayernor, G. S., Truong, V. D., Shih, F. F., & Daigle, K. (2014). Variability of sugars in staple-type sweet potato (Ipomoea batatas) cultivars: the effects of harvest time and storage. *International Journal of Food Properties*, *17*(2), 410-420.

Agarwal, D., Mui, L., Aldridge, E., McKinney, J., Hewson, L., & Fisk, I. D. (2021). The progression of lipid oxidation, β-carotenes degradation and sensory perception of batch-fried sliced sweet potato crisps during storage. *Food & Function*, *12*(10), 4535–4543. <https://doi.org/10.1039/D0FO03100C>

Aisman, Syukri, D., Rini, Jaswandi, J., & Haiyee, Z. A. (2024). Optimum Condition for the Formation of β-ionone by Thermal Decomposition of Carotenoids Extract from Orange Sweet Potato (Ipomoea Batatas L.). *The Open Agriculture Journal*, *18*(1), e18743315300431. <https://doi.org/10.2174/0118743315300431240415103610>

Allan, M. C., Johanningsmeier, S. D., Nakitto, M., Guambe, O., Abugu, M., Pecota, K. V., & Craig Yencho, G. (2024). Baked sweetpotato textures and sweetness: An investigation into relationships between physicochemical and cooked attributes. *Food Chemistry: X*, *21*, 101072. <https://doi.org/10.1016/j.fochx.2023.101072>

Allan, M. C., Read, Q. D., & Johanningsmeier, S. D. (2023). Impact of sweetpotato starch structures, thermal properties, and granules sizes on sweetpotato fry textures. *Food Hydrocolloids*, *137*, 108377. <https://doi.org/10.1016/j.foodhyd.2022.108377>

Ayala, J. A. C., Olivas, A. F., Soto, J. H. V., Pagaza, Y. R., Castillo, F. D. H., López, P. F., ... & Chávez, E. R. (2020). Preference for Oviposition by Sweetpotato Whitefly, Bemisia tabaci (Gennadius) 1, in Two Soybean Genotypes, and Volatile Release. *Southwestern Entomologist*, *45*(1), 99-108.

Baafi, E., Gracen, V. E., Manu-Aduening, J., Blay, E. T., Ofori, K., & Carey, E. E. (2017). Genetic control of dry matter, starch and sugar content in sweetpotato. *Acta Agriculturae Scandinavica, Section B—Soil & Plant Science*, *67*(2), 110-118.

Baba, T., NAKAMA, H., TAMARU, Y., & KONO, T. (1987). Changes in Sugar and Starch Contents During Storage of New Type Sweet Potato (Low β-Amylase Activity in Roots) Development of Snack Foods Producted from Sweet Potatoes Part V. *Nippon shokuhin kogyo gakkaishi*, *34*(4), 249-253.

Barkley, S. L., Schultheis, J. R., Chaudhari, S., Johanningsmeier, S. D., Jennings, K. M., Truong, V.-D., & Monks, D. W. (2017). Yield and Consumer Acceptability of ‘Evangeline’ Sweetpotato for Production in North Carolina. *HortTechnology*, *27*(2), 281–290. <https://doi.org/10.21273/HORTTECH03533-16>

Bechoff, A., Dhuique-Mayer, C., Dornier, M., Tomlins, K. I., Boulanger, R., Dufour, D., & Westby, A. (2010). Relationship between the kinetics of β-carotene degradation and formation of norisoprenoids in the storage of dried sweet potato chips. *Food Chemistry*, *121*(2), 348–357. <https://doi.org/10.1016/j.foodchem.2009.12.035>

Bordiga, M., & Nollet, L. M. L. (Eds.). (2019). *Food aroma evolution: During food processing, cooking and aging* (1st edition). CRC Press.

Caetano, P. K., Mariano-Nasser, F. A. D. C., Mendonça, V. Z. D., Furlaneto, K. A., Daiuto, E. R., & Vieites, R. L. (2017). Physicochemical and sensory characteristics of sweet potato chips undergoing different cooking methods. *Food Science and Technology*, *38*(3), 434–440. <https://doi.org/10.1590/1678-457x.08217>

Cao, X., Su, Y., Zhao, T., Zhang, Y., Cheng, B., Xie, K., Yu, M., Allan, A., Klee, H., Chen, K., Guan, X., Zhang, Y., & Zhang, B. (2024). Multi-omics analysis unravels chemical roadmap and genetic basis for peach fruit aroma improvement. *Cell Reports*, *43*(8), 114623. <https://doi.org/10.1016/j.celrep.2024.114623>

Cartier, A., Woods, J., Sismour, E., Allen, J., Ford, E., Githinji, L., & Xu, Y. (2017). Physiochemical, nutritional and antioxidant properties of fourteen Virginia-grown sweet potato varieties. *Journal of Food Measurement and Characterization*, *11*(3), 1333–1341. <https://doi.org/10.1007/s11694-017-9511-8>

Cassilly, J. P. (1971). *AN ATTEMPT TO ISOLATE THE PRECURSORS OF OFF FLAVOR IN OXIDIZED DEHYDRATEDSWEET POTATO FLAKES*. The University of North Carolina at Greensboro.

Cervantes-Flores, J. C., Sosinski, B., Pecota, K. V., Mwanga, R. O. M., Catignani, G. L., Truong, V. D., Watkins, R. H., Ulmer, M. R., & Yencho, G. C. (2011). Identification of quantitative trait loci for dry-matter, starch, and β-carotene content in sweetpotato. *Molecular Breeding*, *28*(2), 201–216. <https://doi.org/10.1007/s11032-010-9474-5>

Chan, C. F., Chiang, C. M., Lai, Y. C., Huang, C. L., Kao, S. C., & Liao, W. C. (2014). Changes in sugar composition during baking and their effects on sensory attributes of baked sweet potatoes. *Journal of food science and technology*, *51*, 4072-4077.

Chan, C.-F., Chiang, C.-M., Lai, Y.-C., Huang, C.-L., Kao, S.-C., & Liao, W. C. (2014). Changes in sugar composition during baking and their effects on sensory attributes of baked sweet potatoes. *Journal of Food Science and Technology*, *51*(12), 4072–4077. <https://doi.org/10.1007/s13197-012-0900-z>

Chapman Jr, G. W., & Horvat, R. J. (1989). Determination of nonvolatile acids and sugars from fruits and sweet potato extracts by capillary GLC and GLC/MS. *Journal of Agricultural and Food Chemistry*, *37*(4), 947-950.

Chedea, V. S., & Jisaka, M. (2013). Lipoxygenase and carotenoids: A co-oxidation story. *African Journal of Biotechnology*, *12*(20).

Chen, F., Ro, D.-K., Petri, J., Gershenzon, J., Bohlmann, J., Pichersky, E., & Tholl, D. (2004). Characterization of a Root-Specific Arabidopsis Terpene Synthase Responsible for the Formation of the Volatile Monoterpene 1,8-Cineole. *Plant Physiology*, *135*(4), 1956–1966. <https://doi.org/10.1104/pp.104.044388>

Cho, S. A., & Yoo, B. (2010). Comparison of the effect of sugars on the viscoelastic properties of sweet potato starch pastes. *International Journal of Food Science and Technology*, *45*(2), 410-414.

Cho, S. A., Kim, B. Y., & Yoo, B. S. (2008). Flow behavior of sweet potato starch in mixed sugar systems. *Preventive Nutrition and Food Science*, *13*(3), 249-252.

Chu, F. L., & Yaylayan, V. A. (2008). Model Studies on the Oxygen-Induced Formation of Benzaldehyde from Phenylacetaldehyde Using Pyrolysis GC-MS and FTIR. *Journal of Agricultural and Food Chemistry*, *56*(22), 10697–10704. <https://doi.org/10.1021/jf8022468>

Clark, C. A. (1989). Influence of volatiles from healthy and decaying sweet potato storage roots on sclerotial germination and hyphal growth of Sclerotium rolfsii. *Canadian journal of botany*, *67*(1), 53-57.

Colantonio, V., Ferrão, L. F. V., Tieman, D. M., Bliznyuk, N., Sims, C., Klee, H. J., Munoz, P., & Resende, M. F. R. (2022). Metabolomic selection for enhanced fruit flavor. *Proceedings of the National Academy of Sciences*, *119*(7), e2115865119. <https://doi.org/10.1073/pnas.2115865119>

Da Silva Pereira, G., Gemenet, D. C., Mollinari, M., Olukolu, B. A., Wood, J. C., Diaz, F., Mosquera, V., Gruneberg, W. J., Khan, A., Buell, C. R., Yencho, G. C., & Zeng, Z.-B. (2020). Multiple QTL Mapping in Autopolyploids: A Random-Effect Model Approach with Application in a Hexaploid Sweetpotato Full-Sib Population. *Genetics*, *215*(3), 579–595. <https://doi.org/10.1534/genetics.120.303080>

De Albuquerque, T. M. R., Sampaio, K. B., & De Souza, E. L. (2019). Sweet potato roots: Unrevealing an old food as a source of health promoting bioactive compounds – A review. *Trends in Food Science & Technology*, *85*, 277–286. <https://doi.org/10.1016/j.tifs.2018.11.006>

Dery, E. K., Carey, E. E., Ssali, R. T., Low, J. W., Johanningsmeier, S. D., Oduro, I., Boakye, A., Omodamiro, R. M., & Yusuf, H. L. (2021). Sensory characteristics and consumer segmentation of fried sweetpotato for expanded markets in Africa. *International Journal of Food Science & Technology*, *56*(3), 1419–1431. <https://doi.org/10.1111/ijfs.14847>

Dincer, C., Karaoglan, M., Erden, F., Tetik, N., Topuz, A., & Ozdemir, F. (2011). Effects of baking and boiling on the nutritional and antioxidant properties of sweet potato [Ipomoea batatas (L.) Lam.] cultivars. *Plant Foods for Human Nutrition*, *66*, 341-347.

Dini, I., Tenore, G. C., Trimarco, E., & Dini, A. (2006). Seven new aminoacyl sugars in Ipomoea batatas. *Journal of agricultural and food chemistry*, *54*(16), 6089-6093.

Drake, M. A., & Civille, G. V. (2003). Flavor Lexicons. *Comprehensive Reviews in Food Science and Food Safety*, *2*(1), 33–40. <https://doi.org/10.1111/j.1541-4337.2003.tb00013.x>

Drapal, M., & Fraser, P. D. (2019). Determination of carotenoids in sweet potato (Ipomoea batatas L., Lam) tubers: Implications for accurate provitamin A determination in staple sturdy tuber crops. *Phytochemistry*, *167*, 112102. <https://doi.org/10.1016/j.phytochem.2019.112102>

Drapal, M., Rossel, G., Heider, B., & Fraser, P. D. (2019). Metabolic diversity in sweet potato (Ipomoea batatas, Lam.) leaves and storage roots. *Horticulture Research*, *6*(1), 2. <https://doi.org/10.1038/s41438-018-0075-5>

Dudareva, N., Klempien, A., Muhlemann, J. K., & Kaplan, I. (2013). Biosynthesis, function and metabolic engineering of plant volatile organic compounds. *New Phytologist*, *198*(1), 16–32. <https://doi.org/10.1111/nph.12145>

Dumas, J. A., Ortiz, C. E., & Soler, S. M. (2005). Relationship between sweet potato physical properties and volatiles release during baking. <https://doi.org/10.22004/AG.ECON.264162>

Eggink, P. M., Maliepaard, C., Tikunov, Y., Haanstra, J. P. W., Pohu-Flament, L. M. M., De Wit-Maljaars, S. C., Willeboordse-Vos, F., Bos, S., Benning-de Waard, C., De Grauw-van Leeuwen, P. J., Freymark, G., Bovy, A. G., & Visser, R. G. F. (2012). Prediction of sweet pepper (Capsicum annuum) flavor over different harvests. *Euphytica*, *187*(1), 117–131. <https://doi.org/10.1007/s10681-012-0761-6>

Fan, Z., Tieman, D. M., Knapp, S. J., Zerbe, P., Famula, R., Barbey, C. R., Folta, K. M., Amadeu, R. R., Lee, M., Oh, Y., Lee, S., & Whitaker, V. M. (2022). A multi‐omics framework reveals strawberry flavor genes and their regulatory elements. *New Phytologist*, *236*(3), 1089–1107. <https://doi.org/10.1111/nph.18416>

Feng, J., Berton-Carabin, C. C., Fogliano, V., & Schroën, K. (2022). Maillard reaction products as functional components in oil-in-water emulsions: A review highlighting interfacial and antioxidant properties. *Trends in Food Science & Technology*, *121*, 129-141. <https://doi.org/10.1016/j.tifs.2022.02.008>

Firon, N., LaBonte, D., Villordon, A., Kfir, Y., Solis, J., Lapis, E., Perlman, T., Doron-Faigenboim, A., Hetzroni, A., Althan, L., & Nadir, L. (2013). Transcriptional profiling of sweetpotato (Ipomoea batatas) roots indicates down-regulation of lignin biosynthesis and up-regulation of starch biosynthesis at an early stage of storage root formation. *BMC Genomics*, *14*(1), 460. <https://doi.org/10.1186/1471-2164-14-460>

Fu, Y., Zhang, Y., Soladoye, O. P., & Aluko, R. E. (2020). Maillard reaction products derived from food protein-derived peptides: Insights into flavor and bioactivity. *Critical Reviews in Food Science and Nutrition*, *60*(20), 3429–3442. <https://doi.org/10.1080/10408398.2019.1691500>

García-Martínez, M. C., Márquez-Ruiz, G., Fontecha, J., & Gordon, M. H. (2009). Volatile oxidation compounds in a conjugated linoleic acid-rich oil. *Food Chemistry*, *113*(4), 926–931. <https://doi.org/10.1016/j.foodchem.2008.08.020>

Gemenet, D. C., Da Silva Pereira, G., De Boeck, B., Wood, J. C., Mollinari, M., Olukolu, B. A., Diaz, F., Mosquera, V., Ssali, R. T., David, M., Kitavi, M. N., Burgos, G., Felde, T. Z., Ghislain, M., Carey, E., Swanckaert, J., Coin, L. J. M., Fei, Z., Hamilton, J. P., … Buell, C. R. (2020). Quantitative trait loci and differential gene expression analyses reveal the genetic basis for negatively associated β-carotene and starch content in hexaploid sweetpotato [Ipomoea batatas (L.) Lam.]. *Theoretical and Applied Genetics*, *133*(1), 23–36. <https://doi.org/10.1007/s00122-019-03437-7>

Gemenet, D. C., Kitavi, M. N., David, M., Ndege, D., Ssali, R. T., Swanckaert, J., Makunde, G., Yencho, G. C., Gruneberg, W., Carey, E., Mwanga, R. O., Andrade, M. I., Heck, S., & Campos, H. (2020). Development of diagnostic SNP markers for quality assurance and control in sweetpotato [Ipomoea batatas (L.) Lam.] breeding programs. *PLOS ONE*, *15*(4), e0232173. <https://doi.org/10.1371/journal.pone.0232173>

Gong, C., He, N., Zhu, H., Anees, M., Lu, X., & Liu, W. (2023). Multi-omics integration to explore the molecular insight into the volatile organic compounds in watermelon. *Food Research International*, *166*, 112603. <https://doi.org/10.1016/j.foodres.2023.112603>

Grebenteuch, S., Kroh, L. W., Drusch, S., & Rohn, S. (2021). Formation of Secondary and Tertiary Volatile Compounds Resulting from the Lipid Oxidation of Rapeseed Oil. *Foods*, *10*(10), 2417. <https://doi.org/10.3390/foods10102417>

Guo, Q., & Mu, T. H. (2011). Emulsifying properties of sweet potato protein: Effect of protein concentration and oil volume fraction. *Food Hydrocolloids*, *25*(1), 98–106. <https://doi.org/10.1016/j.foodhyd.2010.05.011>

Habinshuti, I., Mu, T.-H., & Zhang, M. (2021). Structural, antioxidant, aroma, and sensory characteristics of Maillard reaction products from sweet potato protein hydrolysates as influenced by different ultrasound-assisted enzymatic treatments. *Food Chemistry*, *361*, 130090. <https://doi.org/10.1016/j.foodchem.2021.130090>

Hagenimana, V., Vézina, L., & Simard, R. E. (1994). Sweetpotato α‐ and β‐Amylases: Characterization and Kinetic Studies with Endogenous Inhibitors. *Journal of Food Science*, *59*(2), 373–376. <https://doi.org/10.1111/j.1365-2621.1994.tb06970.x>

He, H. J., Wang, Y., Zhang, M., Wang, Y., Ou, X., & Guo, J. (2022). Rapid determination of reducing sugar content in sweet potatoes using NIR spectra. *Journal of Food Composition and Analysis*, *111*, 104641.

Hemmler, D., Roullier-Gall, C., Marshall, J. W., Rychlik, M., Taylor, A. J., & Schmitt-Kopplin, P. (2017). Evolution of Complex Maillard Chemical Reactions, Resolved in Time. *Scientific Reports*, *7*(1), 3227. <https://doi.org/10.1038/s41598-017-03691-z>

Hemmler, D., Roullier-Gall, C., Marshall, J. W., Rychlik, M., Taylor, A. J., & Schmitt-Kopplin, P. (2018). Insights into the Chemistry of Non-Enzymatic Browning Reactions in Different Ribose-Amino Acid Model Systems. *Scientific Reports*, *8*(1), 16879. <https://doi.org/10.1038/s41598-018-34335-5>

Henderson, S. K., & Nawar, W. W. (1981). Thermal interaction of linoleic acid and its esters with valine. *Journal of the American Oil Chemists’ Society*, *58*(5), 632–635. <https://doi.org/10.1007/BF02672381>

Hernandez-Carrion, T., Ortiz, C. E., Montalvo-Zapata, R., & Rivera, L. E. (2010). SUGARS IN TROPICAL-TYPE SWEET POTATO VARIETIES OF PUERTO RICO1-2.

Hernández-Carrión, T., Ortiz, C. E., Montalvo-Zapata, R., & Rivera, L. E. (2011). Sugars and alcohol insoluble solids assessment for sweet potato cultivars recommended for Puerto Rico. *J. Agric Univ PR*, *95*, 223-231.

Hidalgo, F. J., Alcón, E., & Zamora, R. (2013). Cysteine- and serine-thermal degradation products promote the formation of Strecker aldehydes in amino acid reaction mixtures. *Food Research International*, *54*(2), 1394–1399. <https://doi.org/10.1016/j.foodres.2013.09.006>

Hitimana, N., McKinlay, R. G., & Hunter, E. A. (2000). Attractiveness of host plant volatiles to sweet potato butterfly Acraea acerata (Lepidoptera Nymphalidae).

Hofmann, T., & Schieberle, P. (2000). Formation of Aroma-Active Strecker-Aldehydes by a Direct Oxidative Degradation of Amadori Compounds. *Journal of Agricultural and Food Chemistry*, *48*(9), 4301–4305. <https://doi.org/10.1021/jf000076e>

Horvat, R. J., Arrendale, R. F., Dull, G. G., Chapman Jr, G. W., & Kays, S. J. (1991). Volatile constituents and sugars of three diverse cultivars of sweet potatoes [Ipomoea batatas (L.) Lam.]. *Journal of food science*, *56*(3), 714-715.

Horvat, R. J., Arrendale, R. F., Dull, G. G., Chapman, G. W., & Kays, S. J. (1991). Volatile Constituents and Sugars of Three Diverse Cultivars of Sweet Potatoes [Ipomoea batatas (L.) Lam.]. *Journal of Food Science*, *56*(3), 714–715. <https://doi.org/10.1111/j.1365-2621.1991.tb05363.x>

Hou, F., Mu, T., Ma, M., & Blecker, C. (2020). Sensory evaluation of roasted sweet potatoes influenced by different cultivars: A correlation study with respect to sugars, amino acids, volatile compounds, and colors. *Journal of Food Processing and Preservation*, *44*(9). <https://doi.org/10.1111/jfpp.14646>

Hu, J. S., Gelman, D. B., Salvucci, M. E., Chen, Y. P., & Blackburn, M. B. (2010). Insecticidal activity of some reducing sugars against the sweet potato whitefly, Bemisia tabaci, Biotype B. *Journal of Insect Science*, *10*(1), 203.

Hua, J., Fu, Y., Zhou, Q., Huang, Y., Li, H., Chen, T., ... & Li, Z. (2021). Three chemosensory proteins from the sweet potato weevil, Cylas formicarius, are involved in the perception of host plant volatiles. *Pest Management Science*, *77*(10), 4497-4509.

Hua, J., Pan, C., Huang, Y., Li, Y., Li, H., Wu, C., ... & Li, Z. (2021). Functional characteristic analysis of three odorant‐binding proteins from the sweet potato weevil (Cylas formicarius) in the perception of sex pheromones and host plant volatiles. *Pest Management Science*, *77*(1), 300-312.

Huang, Y. H., Picha, D. H., Kilili, A. W., & Johnson, C. E. (1999). Changes in invertase activities and reducing sugar content in sweetpotato stored at different temperatures. *Journal of Agricultural and Food Chemistry*, *47*(12), 4927-4931.

Inukai, Y., Shibayama, H., & Matsubayashi, T. (2002). Growth conditions affecting palatability, especially sweetness of sweet potato.

Januário, J. G. B., Da Silva, I. C. F., De Oliveira, A. S., De Oliveira, J. F., Dionísio, J. N., Klososki, S. J., & Pimentel, T. C. (2017). Probiotic yoghurt flavored with organic beet with carrot, cassava, sweet potato or corn juice: Physicochemical and texture evaluation, probiotic viability and acceptance. *International Food Research Journal*, *24*(1).

Jia XiaoJian, J. X., Ma Juan, M. J., Gao Bo, G. B., Li XiuHua, L. X., Zhang Tao, Z. T., Chen ShuLong, C. S., & Wang RongYan, W. R. (2017). EAG and olfactory responses of Cylas formicarius (Coleoptera: Curculionidae) to volatiles from plants of different sweetpotato cultivars.

Jiang, G., Zeng, X., Wang, Z., Chen, A., Li, S., Liu, X., ... & Zhang, Z. (2018, September). Flavor And Stability Improvement Of Purple Sweet Potato-Peanut Compound Beverage. In *IOP Conference Series: Earth and Environmental Science* (Vol. 186, No. 6, p. 012020). IOP Publishing.

Jiang, X., Zhang, R., Yao, Y., Yang, Y., Wang, B., & Wang, Z. (2023). Effect of cooking methods on metabolites of deep purple-fleshed sweetpotato. *Food Chemistry*, *429*, 136931. <https://doi.org/10.1016/j.foodchem.2023.136931>

Josephson, D. B., & Lindsay, R. C. (1987). c4‐Heptenal: An Influential Volatile Compound in Boiled Potato Flavor. *Journal of Food Science*, *52*(2), 328–331. <https://doi.org/10.1111/j.1365-2621.1987.tb06605.x>

Kadowaki, M., Kubota, F., & Saitou, K. (2001). Effects of exogenous injection of different sugars on leaf photosynthesis, dry matter production and adenosine 5′‐diphosphate glucose pyrophosphorylase (AGPase) activity in sweet potato, Ipomoea batatas (Lam.). *Journal of Agronomy and Crop Science*, *186*(1), 37-41.

Kanasawud, P., & Crouzet, J. C. (1990). Mechanism of formation of volatile compounds by thermal degradation of carotenoids in aqueous medium. 2. Lycopene degradation. *Journal of Agricultural and Food Chemistry*, *38*(5), 1238–1242. <https://doi.org/10.1021/jf00095a018>

Kanzler, C., Schestkowa, H., Haase, P. T., & Kroh, L. W. (2017). Formation of Reactive Intermediates, Color, and Antioxidant Activity in the Maillard Reaction of Maltose in Comparison to d -Glucose. *Journal of Agricultural and Food Chemistry*, *65*(40), 8957–8965. <https://doi.org/10.1021/acs.jafc.7b04105>

Karuniawan, A., Maulana, H., Anindita, P. A., Yoel, A., Ustari, D., Suganda, T., & Concibido, V. (2021). Storage root yield and sweetness level selection for new honey sweet potato (Ipomoea batatas [L.] Lam). *Open Agriculture*, *6*(1), 329-345.

Katayama, K., Komaki, K., & Tamiya, S. (1996). Prediction of starch, moisture, and sugar in sweet potato by near infrared transmittance.

Kaur, G., Abugu, M., & Tieman, D. (2023). The dissection of tomato flavor: Biochemistry, genetics, and omics. *Frontiers in Plant Science*, *14*, 1144113. <https://doi.org/10.3389/fpls.2023.1144113>

Kays, S. J. (2018). Formulated sweet potato products. In *Sweet Potato Products* (pp. 205-218). CRC Press.

Kays, S. J., & Wang, Y. (2002). Sweetpotato quality: its importance, assessment and selection in breeding programs. *Acta Horticulturae*, *583*, 187–193. <https://doi.org/10.17660/ActaHortic.2002.583.21>

Kays, S. J., Wang, Y., & McLaurin, W. J. (1998). Development of alternative flavour types of sweetpotato as a means of expanding consumption.

Kays, S. J., Wang, Y., & McLaurin, W. J. (2005). Chemical and geographical assessment of the sweetness of the cultivated sweetpotato clones of the world. *Journal of the American Society for Horticultural Science*, *130*(4), 591-597.

Kim, H. S., Wang, W., Kang, L., Kim, S.-E., Lee, C.-J., Park, S.-C., Park, W. S., Ahn, M.-J., & Kwak, S.-S. (2020). Metabolic engineering of low-molecular-weight antioxidants in sweetpotato. *Plant Biotechnology Reports*, *14*(2), 193–205. <https://doi.org/10.1007/s11816-020-00621-w>

Kim, H. W., Kim, J. B., Poovan, S., Chung, M. N., Cho, S. M., Lee, Y. M., Cho, Y. S., Kim, J. H., & Kim, H. R. (2014). Effect of processing conditions on the content of *cis* / *trans* carotene isomers as provitamin A carotenoids in Korean sweet potato varieties. *International Journal of Food Sciences and Nutrition*, *65*(7), 821–826. <https://doi.org/10.3109/09637486.2013.854742>

Kim, M. H., Yoshitake, K., Takamine, K., Lee, H. U., & Kim, W. S. (2015). Aromatic ingredients and distinct flavors of the koguma-Soju produced from Korean sweet potato varieties yeonmi, jeungmi, shincheonmi, and shinyeulmi. *Korean Journal of Food Science and Technology*, *47*(1), 51-55.

Koehler, P. E., & Kays, S. J. (1991). Sweet potato flavor: quantitative and qualitative assessment of optimum sweetness. *Journal of food quality*, *14*(3), 241-249.

Koehler, P. E., & Kays, S. J. (1991). Sweet potato flavor: quantitative and qualitative assessment of optimum sweetness. *Journal of Food Quality*, *14*(3), 241–249. <https://doi.org/10.1111/j.1745-4557.1991.tb00065.x>

Kohyama, K., & Nishinari, K. (1991). Effect of soluble sugars on gelatinization and retrogradation of sweet potato starch. *Journal of Agricultural and Food Chemistry*, *39*(8), 1406-1410.

Korada, R. R., Misra, S., Naskar, S. K., Bhaktavatsalam, N., Prasad, A. R., Sinha, K., ... & Mukherjee, A. (2013). Plant volatile organic compounds as chemical markers to identify resistance in sweet potato against weevil Cylas formicarius. *Current Science*, 1247-1253.

Korada, R. R., Naskar, S. K., Prasad, A. R., Prasuna, A. L., & Jyothi, K. N. (2010). Differential volatile emission from sweet potato plant: mechanism of resistance in sweet potato for weevil Cylas formicarius (Fab.). *Current Science*, 1597-1601.

Kouassi, A. B., Durel, C.-E., Costa, F., Tartarini, S., Van De Weg, E., Evans, K., Fernandez-Fernandez, F., Govan, C., Boudichevskaja, A., Dunemann, F., Antofie, A., Lateur, M., Stankiewicz-Kosyl, M., Soska, A., Tomala, K., Lewandowski, M., Rutkovski, K., Zurawicz, E., Guerra, W., & Laurens, F. (2009). Estimation of genetic parameters and prediction of breeding values for apple fruit-quality traits using pedigreed plant material in Europe. *Tree Genetics & Genomes*, *5*(4), 659–672. <https://doi.org/10.1007/s11295-009-0217-x>

Lai, Y. C., Huang, C. L., Chan, C. F., Lien, C. Y., & Liao, W. C. (2013). Studies of sugar composition and starch morphology of baked sweet potatoes (Ipomoea batatas (L.) Lam). *Journal of Food Science and Technology*, *50*, 1193-1199.

Lancioni, C., Castells, C., Candal, R., & Tascon, M. (2022). Headspace solid-phase microextraction: Fundamentals and recent advances. *Advances in Sample Preparation*, *3*, 100035. <https://doi.org/10.1016/j.sampre.2022.100035>

Laurie, S. M., Faber, M., Calitz, F. J., Moelich, E. I., Muller, N., & Labuschagne, M. T. (2013). The use of sensory attributes, sugar content, instrumental data and consumer acceptability in selection of sweet potato varieties. *Journal of the Science of Food and Agriculture*, *93*(7), 1610-1619.

Laveriano-Santos, E. P., López-Yerena, A., Jaime-Rodríguez, C., González-Coria, J., Lamuela-Raventós, R. M., Vallverdú-Queralt, A., Romanyà, J., & Pérez, M. (2022). Sweet Potato Is Not Simply an Abundant Food Crop: A Comprehensive Review of Its Phytochemical Constituents, Biological Activities, and the Effects of Processing. *Antioxidants*, *11*(9), 1648. <https://doi.org/10.3390/antiox11091648>

Lebot, V. (2017). Rapid quantitative determination of maltose and total sugars in sweet potato (Ipomoea batatas L.[Lam.]) varieties using HPTLC. *Journal of food science and technology*, *54*(3), 718-726.

Lee, S. J., Kwon, H. S., Shin, W. C., Choi, J. Y., & Noh, B. S. (2018). Analysis of the flavor of aged spirits made from sweet potato and rice by gas chromatography–mass spectrometry. *Food science and biotechnology*, *27*, 313-322.

Leighton, C. S., Schönfeldt, H. C., & Kruger, R. (2010). Quantitative descriptive sensory analysis of five different cultivars of sweet potato to determine sensory and textural profiles. *Journal of Sensory Studies*, *25*(1), 2–18. <https://doi.org/10.1111/j.1745-459X.2008.00188.x>

Leksrisompong, P. P., Whitson, M. E., Truong, V. D., & Drake, M. A. (2012). Sensory attributes and consumer acceptance of sweet potato cultivars with varying flesh colors: sensory analysis of sweet potatoes. *Journal of Sensory Studies*, *27*(1), 59–69. <https://doi.org/10.1111/j.1745-459X.2011.00367.x>

Lewthwaite, S. L., Sutton, K. H., & Triggs, C. M. (1997). Free sugar composition of sweetpotato cultivars after storage. New Zealand journal of crop and horticultural science, 25(1), 33-41.

Lewthwaite, S. L., Triggs, C. M., & Sutton, K. H. (2000). Preliminary study on sweet potato growth: II. Sugar composition of developing storage roots.

Li, C. U. I., LIU, C. Q., & LI, D. J. (2010). Changes in volatile compounds of sweet potato tips during fermentation. *Agricultural Sciences in China*, *9*(11), 1689-1695.

Li, S., An, Y., Fu, W., Sun, X., Li, W., & Li, T. (2017). Changes in anthocyanins and volatile components of purple sweet potato fermented alcoholic beverage during aging. *Food research international*, *100*, 235-240.

Li, X. D., Li, J., Wang, M., & Jiang, H. (2016). Copigmentation effects and thermal degradation kinetics of purple sweet potato anthocyanins with metal ions and sugars. *Applied Biological Chemistry*, *59*, 15-24.

Li, X., Li, B., Cai, S., Zhang, Y., Xu, M., Zhang, C., ... & Qin, S. (2020). Identification of rhizospheric actinomycete Streptomyces lavendulae sps-33 and the inhibitory effect of its volatile organic compounds against Ceratocystis fimbriata in postharvest sweet potato (Ipomoea batatas (L.) Lam.). *Microorganisms*, *8*(3), 319.

Liu HongJuan, L. H., Li ZuoMei, L. Z., Shi ChunYu, S. C., & Zhang LiMing, Z. L. (2011). Physiological basis of improving soluble sugar content in sweetpotato for table use by humic acid application.

Liu, H. J., Shi, C. Y., Zhang, L. M., Zhang, H. F., Wang, Z. Z., & Chai, S. S. (2012). Effect of potassium on the activities of enzymes related to sugar metabolism of edible sweet potato. *JOURNAL OF PLANT NUTRITION AND FERTILIZER*.

Liu, S., Sun, H., Ma, G., Zhang, T., Wang, L., Pei, H., Li, X., & Gao, L. (2022). Insights into flavor and key influencing factors of Maillard reaction products: A recent update. *Frontiers in Nutrition*, *9*, 973677. <https://doi.org/10.3389/fnut.2022.973677>

Liu, Y., Zhang, T., Zheng, M. Z., Liu, H. M., & Zhang, Y. (2020). Relationship between sugar content in sweet potato and vibration acoustic signal eigenvalues. *Journal of Food Process Engineering*, *43*(4), e13359.

Longin, F., Beck, H., Gütler, H., Heilig, W., Kleinert, M., Rapp, M., Philipp, N., Erban, A., Brilhaus, D., Mettler-Altmann, T., & Stich, B. (2020). Aroma and quality of breads baked from old and modern wheat varieties and their prediction from genomic and flour-based metabolite profiles. *Food Research International*, *129*, 108748. <https://doi.org/10.1016/j.foodres.2019.108748>

Maeo, K., Tomiya, T., Hayashi, K., Akaike, M., Morikami, A., Ishiguro, S., & Nakamura, K. (2001). Sugar-responsible elements in the promoter of a gene for β-amylase of sweet potato. *Plant molecular biology*, *46*, 627-637.

Maeo, K., Tomiya, T., Hayashi, K., Akaike, M., Morikami, A., Ishiguro, S., & Nakamura, K. (2001). Sugar-responsible elements in the promoter of a gene for β-amylase of sweet potato. *Plant molecular biology*, *46*, 627-637.

Mandin, O., Duckham, S. C., & Ames, J. M. (1999). Volatile Compounds from Potato-like Model Systems. *Journal of Agricultural and Food Chemistry*, *47*(6), 2355–2359. <https://doi.org/10.1021/jf981277>

Marques, T. S., Moreira, R. F. A., & Ayres, E. M. M. (2022). Characterization of the essential oils from leaves of different sweet potato cultivars grown in Brazil. *South African Journal of Botany*, *144*, 18–22. <https://doi.org/10.1016/j.sajb.2021.09.005>

Martin, F. W., & Deshpande, S. N. (1985). Sugars and starches in a non-sweet sweetpotato compared to those of conventional cultivars. *J. Agric. Univ. Puerto Rico*, *69*(3), 401.

Martin, F. W., & Rodriguez-Sosa, E. J. (1985). Preference for color, sweetness, and mouthfeel of sweet potato in Puerto Rico. *Journal of the University of Puerto-Rico*, *69*, 99-106.

Martins, S. I. F. S., Jongen, W. M. F., & Van Boekel, M. A. J. S. (2000). A review of Maillard reaction in food and implications to kinetic modelling. *Trends in Food Science & Technology*, *11*(9–10), 364–373. <https://doi.org/10.1016/S0924-2244(01)00022-X>

Mateus, M. M., Ventura, P., Rego, A., Motta, C., Castanheira, I., Bordado, J. M., & dos Santos, R. G. (2017). Acid liquefaction of potato (Solanum tuberosum) and sweet potato (Ipomoea batatas) cultivars peels–Pre-screening of antioxidant Aactivity total phenolic and sugar contents. *BioResources*, *12*(1), 1463-1478.

Mcclements, D., & Decker, E. (2008). Lipids. *Food Chemistry*, 155–216.

McDonald, R. E., & Newson, D. W. (1970). Extraction and Gas-liquid Chromatography of Sweet Potato Sugars and Inositol1. *Journal of the American Society for Horticultural Science*, *95*(3), 299-301.

Meents, A. K., Chen, S. P., Reichelt, M., Lu, H. H., Bartram, S., Yeh, K. W., & Mithöfer, A. (2019). Volatile DMNT systemically induces jasmonate-independent direct anti-herbivore defense in leaves of sweet potato (Ipomoea batatas) plants. *Scientific Reports*, *9*(1), 17431.

Mensah, E. O., Ibok, O., Ellis, W. O., & Carey, E. E. (2016). Thermal stability of β-amylase activity and sugar profile of sweet-potato varieties during processing. *J. Nutr. Food Sci*, *6*, 515.

Mingyu, L. I. U., Limin, C. H. E. N., Sidan, W. A. N. G., Weiqin, D. E. N. G., & Shuliang, L. I. U. (2016). Development and flavor substances analysis of purple sweet potato and bitter buckwheat combined vinegar beverage. *Food and Machinery*, *32*(11), 178-182.

Mondello, L., Tranchida, P. Q., Dugo, P., & Dugo, G. (2008). Comprehensive two‐dimensional gas chromatography‐mass spectrometry: A review. *Mass Spectrometry Reviews*, *27*(2), 101–124. <https://doi.org/10.1002/mas.20158>

Mortley, D. G., Bonsi, C. K., Hill, W. A., Morris, C. E., Williams, C. S., Davis, C. F., ... & Wheeler, R. M. (2008). Influence of microgravity environment on root growth, soluble sugars, and starch concentration of sweetpotato stem cuttings. *Journal of the American Society for Horticultural Science*, *133*(3), 327-332.

Motsa, N. M., Modi, A. T., & Mabhaudhi, T. (2015). Influence of agro-ecological production areas on antioxidant activity, reducing sugar content, and selected phytonutrients of orange-fleshed sweet potato cultivars. *Food Science and Technology (Campinas)*, *35*(1), 32-37.

Muhanna, M., & Rees, D. (2004). The role of root sugar content on the susceptibility of sweetpotato cultivars to soft rot. *African Crop Science Journal*, *12*(3), 305-309.

Nagata, T., & Saitou, K. (2009). Regulation of expression of D3-type cyclins and ADP-glucose pyrophosphorylase genes by sugar, cytokinin and ABA in sweet potato (Ipomoea batatas Lam.). *Plant production science*, *12*(4), 434-442.

Naidoo, S. I., Laurie, S. M., Booyse, M., Mphela, W. M., Makunde, G. S., Shimelis, H., & Laing, M. D. (2021). Combining ability, heterosis and heritability of sweetpotato root protein, β-carotene, sugars and mineral composition. *Euphytica*, *217*(6), 109.

Nakamura, A., Ono, T., Yagi, N., & Miyazawa, M. (2013). Volatile compounds with characteristic aroma of boiled sweet potato (Ipomoea batatas L. cv Ayamurasaki, I. batatas L. cv Beniazuma and I. batatas L. cv Simon 1). *Journal of Essential Oil Research*, *25*(6), 497-505.

Nakamura, A., Ono, T., Yagi, N., & Miyazawa, M. (2013). Volatile compounds with characteristic aroma of boiled sweet potato ( *Ipomoea batatas* L. cv Ayamurasaki, *I*. *Batatas* L. cv Beniazuma and *I. batatas* L. cv Simon 1). *Journal of Essential Oil Research*, *25*(6), 497–505. <https://doi.org/10.1080/10412905.2013.809320>

Nakamura, Y. (2020). Carbohydrate components associated with sweetness of cooked storage roots of sweet potato cultivars.

Nakitto, M., Johanningsmeier, S. D., Moyo, M., Bugaud, C., De Kock, H., Dahdouh, L., Forestier-Chiron, N., Ricci, J., Khakasa, E., Ssali, R. T., Mestres, C., & Muzhingi, T. (2022). Sensory guided selection criteria for breeding consumer-preferred sweetpotatoes in Uganda. *Food Quality and Preference*, *101*, 104628. <https://doi.org/10.1016/j.foodqual.2022.104628>

Ohta, T., Ikuta, R., Nakashima, M., Morimitsu, Y., Samuta, T., & Saiki, H. (1990). Characteristic Flavor of *Kansho-shochu* (Sweet Potato Spirit). *Agricultural and Biological Chemistry*, *54*(6), 1353–1357. <https://doi.org/10.1080/00021369.1990.10870163>

Ohto, M. A., Hayashi, K., Isobe, M., & Nakamura, K. (1995). Involvement of Ca2+ signalling in the sugar‐inducible expression of genes coding for sporamin and β‐amylase of sweet potato. *The Plant Journal*, *7*(2), 297-307.

Owusu-Mensah, E., Oduro, I., Ellis, W. O., & Carey, E. E. (2016). Cooking treatment effects on sugar profile and sweetness of eleven-released sweet potato varieties.

Park, J. S., Chung, B. W., Bae, J. O., Lee, J. H., Jung, M. Y., & Choi, D. S. (2010). Effects of sweet potato cultivars and koji types on general properties and volatile flavor compounds in sweet potato soju. *Korean Journal of Food Science and Technology*, *42*(4), 468-474.

Parker, J. K. (2015). Thermal generation or aroma. In *Flavour Development, Analysis and Perception in Food and Beverages* (pp. 151–185). Elsevier. <https://doi.org/10.1016/B978-1-78242-103-0.00008-4>

Patil, R. R., Khanvilkar, M. H., Kaskar, D. R., & Prabudesai, S. S. (2006). Effect of potassium nutrition on dry matter accumulation, sugars, starch and nutrient concentration in sweet potato.

PEDRAMNIA, A., & Jalali, M. (2021). Optimization of consumption levels of sweet potato puree, inulin and date liquid sugar in order to improve physicochemical and sensory properties of prebiotic ketchup sauce by response surface methodology.

Pereira, C. R., Resende, J. T. V., Guerra, E. P., Lima, V. A., Martins, M. D., & Knob, A. (2017). Enzymatic conversion of sweet potato granular starch into fermentable sugars: Feasibility of sweet potato peel as alternative substrate for α-amylase production. *Biocatalysis and agricultural biotechnology*, *11*, 231-238.

Picha, D. H. (1986). Influence of storage duration and temperature on sweet potato sugar content and chip color. *Journal of Food science*, *51*(1), 239-240.

Pradhan, D. M. P., Gayatree Priyadarshinee, G. P., James George, J. G., Arup Mukherjee, A. M., Pati, K., & Archana Mukherjee, A. M. (2015). High starch, low sugar yielding genotypes of sweet potato and their micropropagation.

Purcell, A. E., & Walter, W. M. (1988). Comparison of carbohydrate components in sweet potatoes baked by convection heating and microwave heating. *Journal of Agricultural and Food Chemistry*, *36*(2), 360–362. <https://doi.org/10.1021/jf00080a029>

Purcell, A. E., Later, D. W., & Lee, M. L. (1980). Analysis of the volatile constituents of baked, “Jewel” sweet potatoes. *Journal of Agricultural and Food Chemistry*, *28*(5), 939–941. <https://doi.org/10.1021/jf60231a033>

QIAO Yu, WANG Rufu*, YU Di, XING Xiaoying, LI Jiangyong, ZHANG Huaimin. Analysis of volatile aroma components in sweet potato vinegar by HS-SPME and GC-MS[J]. *China Brewing*, 2017, 36(7): 178-181 https://doi.org/10.11882/j.issn.0254-5071.2017.07.038

Qiu, X., Reynolds, R., Johanningsmeier, S., & Truong, V.-D. (2020). Determination of free amino acids in five commercial sweetpotato cultivars by hydrophilic interaction liquid chromatography-mass spectrometry. *Journal of Food Composition and Analysis*, *92*, 103522. <https://doi.org/10.1016/j.jfca.2020.103522>

Regueiro, J., Negreira, N., & Simal-Gándara, J. (2017). Challenges in relating concentrations of aromas and tastes with flavor features of foods. *Critical Reviews in Food Science and Nutrition*, *57*(10), 2112–2127. <https://doi.org/10.1080/10408398.2015.1048775>

Rhim, J. W. (2002). Effects of Organic Acids on the Extraction Kinetics of Purple-fleshed Sweet Potato Pigment. *Food Science and Biotechnology*, *11*(4), 346-349.

Rodrigo, D., Jolie, R., Loey, A. V., & Hendrickx, M. (2007). Thermal and high-pressure stability of tomato lipoxygenase and hydroperoxide lyase. *Journal of Food Engineering*, *79*(2), 423–429. <https://doi.org/10.1016/j.jfoodeng.2006.02.005>

Rodriguez, G., Prinyawiwatkul, W., Aryana, K. J., King, J. M., & Xu, Z. (2023). Bound form terpenes in sweet potatoes and their distribution in flesh and peel of different cultivars. *International Journal of Food Science & Technology*, *58*(11), 5773–5780. <https://doi.org/10.1111/ijfs.16675>

Rodríguez, Z., Boucourt, R., Elías, A., & Madera, M. (2001). Dynamic of fermentation of sugar cane (Saccharum officinarum) and sweet potato (Ipomoea batatas) mixtures.

Rodríguez, Z., Elías, A., & Riverí, Z. (1998). Studies on the utilization of sweet potato (Ipomea batatas Lam) in the solid state fermentation of sugar cane. *Cuban Journal of Agricultural Science*, *32*(3), 285-290.

Rolston, L. H., Clark, C. A., Cannon, J. M., Randle, W. M., Riley, E. G., Wilson, P. W., & Robbins, M. L. (1987). Beauregard’ Sweet Potato. *HortScience*, *22*(6), 1338–1339. <https://doi.org/10.21273/HORTSCI.22.6.1338>

Sacchetti, P., Rossi, E., Bellini, L., Vernieri, P., Cioni, P. L., & Flamini, G. (2015). Volatile organic compounds emitted by bottlebrush species affect the behaviour of the sweet potato whitefly. *Arthropod-Plant Interactions*, *9*, 393-403.

Salelign, K., & Duraisamy, R. (2021). Sugar and ethanol production potential of sweet potato (Ipomoea batatas) as an alternative energy feedstock: processing and physicochemical characterizations. *Heliyon*, *7*(11).

Salvador, L. D., Suganuma, T., Kitahara, K., Fukushige, Y., & Tanoue, H. (2002). Degradation of cell wall materials from sweetpotato, cassava, and potato by a bacterial protopectinase and terminal sugar analysis of the resulting solubilized products. *Journal of bioscience and bioengineering*, *93*(1), 64-72.

Samantaray, T., & Korada, R. R. (2016). Electrophysiological and behavioural responses of sweetpotato weevil, Cylas formicarius to green leaf volatiles and terpenoids. *Current Science*, 902-908.

Sawai, J., Nakai, T., Hashimoto, A., & Shimizu, M. (2004). A comparison of the hydrolysis of sweet potato starch with β‐amylase and infrared radiation allows prediction of reducing sugar production. *International journal of food science & technology*, *39*(9), 967-974.

Schwab, W., Fischer, T., & Wüst, M. (2015). Terpene glucoside production: Improved biocatalytic processes using glycosyltransferases. *Engineering in Life Sciences*, *15*(4), 376–386. <https://doi.org/10.1002/elsc.201400156>

Schweinberger, C. M., Trierweiler, J. O., & Trierweiler, L. F. (2019). A simple equation for total reducing sugars (TRS) estimation on sweet potato and ethanol yield potential. *Brazilian Journal of Chemical Engineering*, *36*(1), 33-41.

Shahidi, F., & Hossain, A. (2022). Role of Lipids in Food Flavor Generation. *Molecules*, *27*(15), 5014. <https://doi.org/10.3390/molecules27155014>

Shahidi, F., & Oh, W. Y. (2020). Lipid-derived flavor and off-flavor of traditional and functional foods: An overview. *Journal of Food Bioactives*, 20–31. <https://doi.org/10.31665/JFB.2020.10224>

Sharkey, T. D., Gray, D. W., Pell, H. K., Breneman, S. R., & Topper, L. (2013). Isoprene synthase genes form a monophyletic clade of acyclic terpene synthases in the TPS-B terpene synthase family: isoprene synthase evolution. *Evolution*, *67*(4), 1026–1040. <https://doi.org/10.1111/evo.12013>

Shen, S. F., Xiang, C., Wu, L. H., Li, B., & Luo, Z. G. (2020). Determination and difference analysis of soluble sugar content in 11 sweetpotato germplasm resources.

Shen, S. F., Xiang, C., Wu, L. H., Li, B., & Luo, Z. G. (2021). Analysis on the characteristics of soluble sugar components in sweetpotato storage root and its relationship with taste.

Shen, X., Wang, H., Yao, L., Song, S., Wang, H., Sun, M., Liu, Q., Yu, C., & Feng, T. (2024). Volatile compounds analysis and sensory profiling of four colored roasted sweet potatoes via HS-SPME GC-O-MS and HS-SPME GC×GC-TOF MS. *Journal of Food Composition and Analysis*, *131*, 106256. <https://doi.org/10.1016/j.jfca.2024.106256>

Shunmin, W. A. N. G., Yong, L. I., & Weibing, C. A. O. (2016). Study on process of sugar permeation by microwave vacuum technology in preserved fruit making of low sugar purple sweet potato. *Food and Machinery*, *32*(7), 196-201.

Solihin, M. A., Sitorus, S. R., Sutandi, A., & Widiatmaka, W. (2017). Discriminating land characteristics of yield and total sugar content classes of Cilembu sweet potato (Ipomoea batatas L.). *AGRIVITA Journal of Agricultural Science*, *40*(1), 15-24.

Son, K. C., Severson, R. F., Snook, M. E., & Kays, S. J. (1991). Root carbohydrate, organic acids, and phenolic chemistry in relation to sweetpotato weevil resistance. *HortScience*, *26*(10), 1305-1308.

Srinivasan Damodaran & Kirk L. Parkin. (2017). *Fennema’s Food Chemistry, Fifth Edition*. CRC Press. <https://doi.org/10.1201/9781315372914>

Ssali, R., Carey, E., Imoro, S., Low, J. W., Dery, E. K., Boakye, A., Oduro, I., Omodamiro, R. M., Yusuf, H. L., Etwire, E., Iyilade, A. O., Adekambi, S., Ali, A., Haliru, M., & Etwire, P. M. (2021). Fried sweetpotato user preferences identified in Nigeria and Ghana and implications for trait evaluation. *International Journal of Food Science & Technology*, *56*(3), 1399–1409. <https://doi.org/10.1111/ijfs.14764>

Stilo, F., Bicchi, C., Robbat, A., Reichenbach, S. E., & Cordero, C. (2021). Untargeted approaches in food-omics: The potential of comprehensive two-dimensional gas chromatography/mass spectrometry. *TrAC Trends in Analytical Chemistry*, *135*, 116162. <https://doi.org/10.1016/j.trac.2020.116162>

Stroparo, E. C., Knob, A., de Resende, J. T. V., Schirmer, W. N., & de Álcool, B. C. V. P. (2019). Evaluation of Sweet Potato Cultivars to the Formation of Sugars with Potential for the Production of Ethanol. *Revista Virtual de Química*, *11*(3), 605-615.

Sukhveer Singh, S. S., Singh, U. P., Vishakha Singh, V. S., & Arvind, A. (2017). Formulation and process optimization of phalahari muffin produced from sugar, butter and sweet potato flour.

Sulistiani, R., Siregar, L. A. M., & Harahap, F. (2018, February). Differences in morphology and sugar content of purple sweet potato (Ipomoea batatas L.) with potassium treatment at several altitudes. In *IOP Conference Series: Earth and Environmental Science* (Vol. 122, No. 1, p. 012050). IOP Publishing.

Sun, J., Severson, R. F., & Kays, S. J. (1994). EFFECT OF HEATING TEMPERATURE AND MICROWAVE PRETREATMENT ON THE FORMATION OF SUGARS AND VOLATILES IN JEWEL SWEETPOTATO. *Journal of Food Quality*, *17*(6), 447–456. <https://doi.org/10.1111/j.1745-4557.1994.tb00165.x>

Sun, J.-B., Severson, R. F., Schlotzhauer, W. S., & Kays, S. J. (1995). Identifying Critical Volatiles in the Flavor of Baked `Jewel’ Sweetpotatoes [Ipomoea batatas (L.) Lam.]. *Journal of the American Society for Horticultural Science*, *120*(3), 468–474. <https://doi.org/10.21273/JASHS.120.3.468>

Sun, T., Tadmor, Y., & Li, L. (2020). Pathways for Carotenoid Biosynthesis, Degradation, and Storage. In M. Rodríguez-Concepción & R. Welsch (Eds.), *Plant and Food Carotenoids* (Vol. 2083, pp. 3–23). Springer US. <https://doi.org/10.1007/978-1-4939-9952-1_1>

Sun, W., Zhang, M., Chen, H., Zheng, D., & Fang, Z. (2016). Effects of deodorization on the physicochemical index and volatile compounds of purple sweet potato anthocyanins (PSPAs). *LWT-Food Science and Technology*, *68*, 265-272.

Syukri, D., . R., . W., Rahma Yant, N., & . J. (2021). Production of Aromatic Compounds from Crude Carotene Extract of Carrots by Thermal Degradation: A Preliminary Study. *Asian Journal of Plant Sciences*, *21*(1), 163–168. <https://doi.org/10.3923/ajps.2022.163.168>

TAKAHATA, Y., NODA, T., & NAGATA, T. (1992). Varietal diversity of free sugar composition in storage root of sweet potato. *Japanese journal of breeding*, *42*(3), 515-521.

Tang ZhongHou, T. Z., Li HongMin, L. H., Li Qiang, L. Q., Wei Meng, W. M., Liu ZhengHui, L. Z., & Ding YanFeng, D. Y. (2013). Prediction of starch and sugar contents in sweet potato root by near-infrared spectroscopy (NIRS).

Tang, J., Wang, S.-Q., Hu, K.-D., Huang, Z.-Q., Li, Y.-H., Han, Z., Chen, X.-Y., Hu, L.-Y., Yao, G.-F., & Zhang, H. (2019). Antioxidative capacity is highly associated with the storage property of tuberous roots in different sweetpotato cultivars. *Scientific Reports*, *9*(1), 11141. <https://doi.org/10.1038/s41598-019-47604-8>

Tisarum, R., Theerawitaya, C., Samphumphuang, T., Singh, H. P., & Cha-Um, S. (2020). Foliar application of glycinebetaine regulates soluble sugars and modulates physiological adaptations in sweet potato (Ipomoea batatas) under water deficit. *Protoplasma*, *257*(1), 197-211.

Tiu, C. S., Purcell, A. E., & Collins, W. W. (1985). Contribution of some volatile compounds to sweet potato aroma. *Journal of Agricultural and Food Chemistry*, *33*(2), 223–226. <https://doi.org/10.1021/jf00062a016>

Tomlins, K., Owori, C., Bechoff, A., Menya, G., & Westby, A. (2012). Relationship among the carotenoid content, dry matter content and sensory attributes of sweet potato. *Food Chemistry*, *131*(1), 14–21. <https://doi.org/10.1016/j.foodchem.2011.07.072>

Troise, A. D., Fogliano, V., Vitaglione, P., & Berton-Carabin, C. C. (2020). Interrelated Routes between the Maillard Reaction and Lipid Oxidation in Emulsion Systems. *Journal of Agricultural and Food Chemistry*, *68*(43), 12107–12115. <https://doi.org/10.1021/acs.jafc.0c04738>

Truong, V. D., Avula, R. Y., Pecota, K. V., & Yencho, G. C. (2018). Sweetpotato Production, Processing, and Nutritional Quality. In M. Siddiq & M. A. Uebersax (Eds.), *Handbook of Vegetables and Vegetable Processing* (1st ed., pp. 811–838). Wiley. <https://doi.org/10.1002/9781119098935.ch35>

Tsai, Y.-J., Lin, L.-Y., Yang, K.-M., Chiang, Y.-C., Chen, M.-H., & Chiang, P.-Y. (2021). Effects of Roasting Sweet Potato (Ipomoea batatas L. Lam.): Quality, Volatile Compound Composition, and Sensory Evaluation. *Foods*, *10*(11), 2602. <https://doi.org/10.3390/foods10112602>

USDA-NASS. (2022). *National Agricultural Statistics Service, U.N. USDA/NASS Quick Stats Database.* [Dataset]. <https://quickstats.nass.usda.gov/>

Van Boekel, M. A. J. S. (2006). Formation of flavour compounds in the Maillard reaction. *Biotechnology Advances*, *24*(2), 230–233. <https://doi.org/10.1016/j.biotechadv.2005.11.004>

Van Den, T., Biermann, C. J., & Marlett, J. A. (1986). Simple sugars, oligosaccharides and starch concentrations in raw and cooked sweet potato. *Journal of Agricultural and Food Chemistry*, *34*(3), 421-425.

Van Loon, W. A. M., Linssen, J. P. H., Legger, A., A. Posthumus, M., & Voragen, A. G. J. (2005). Identification and olfactometry of French fries flavour extracted at mouth conditions. *Food Chemistry*, *90*(3), 417–425. <https://doi.org/10.1016/j.foodchem.2004.05.005>

Veeraragavathatham, D., & Jansirani, P. (2006, December). Sweet potato in Indian cusine: use of varieties with the least sweetness. In *I International Conference on Indigenous Vegetables and Legumes. Prospectus for Fighting Poverty, Hunger and Malnutrition 752* (pp. 373-376).

Vincenti, S., Mariani, M., Alberti, J.-C., Jacopini, S., Brunini-Bronzini De Caraffa, V., Berti, L., & Maury, J. (2019). Biocatalytic Synthesis of Natural Green Leaf Volatiles Using the Lipoxygenase Metabolic Pathway. *Catalysts*, *9*(10), 873. <https://doi.org/10.3390/catal9100873>

Wang CuiJuan, W. C., Shi ChunYu, S. C., Liu Na, L. N., Liu ShuangRong, L. S., & Yu XinDi, Y. X. (2016). Comparison of root characteristics and sugar components in root and leaf at early growth phase of sweet potato varieties with significant difference in valid storage root number.

Wang, S., Nie, S., & Zhu, F. (2016). Chemical constituents and health effects of sweet potato. *Food Research International*, *89*, 90–116. <https://doi.org/10.1016/j.foodres.2016.08.032>

Wang, Y., & Kays, S. J. (2000). *Contribution of Volatile Compounds to the Characteristic Aroma of Baked `Jewel’ Sweetpotatoes*. *125*(5), 638–643.

Wang, Y., & Kays, S. J. (2001). Effect of cooking method on the aroma constituents of sweet potatoes [ *ipomoea batatas* (l.) Lam.]. *Journal of Food Quality*, *24*(1), 67–78. <https://doi.org/10.1111/j.1745-4557.2001.tb00591.x>

Wang, Y., & Kays, S. J. (2002). Sweetpotato volatile chemistry in relation to sweetpotato weevil (Cylas formicarius) behavior. *Journal of the American Society for Horticultural Science*, *127*(4), 656-662.

Wei, S., Lu, G., & Cao, H. (2017). Effects of cooking methods on starch and sugar composition of sweetpotato storage roots. *PLoS One*, *12*(8), e0182604.

Wei, S., Lu, G., & Cao, H. (2017). Effects of cooking methods on starch and sugar composition of sweetpotato storage roots. *PLoS One*, *12*(8), e0182604.

Whitfield, F. B., & Mottram, D. S. (1992). Volatiles from interactions of Maillard reactions and lipids. *Critical Reviews in Food Science and Nutrition*, *31*(1–2), 1–58. <https://doi.org/10.1080/10408399209527560>

Winterhalter, P., & Rouseff, R. L. (Eds.). (2002). *Carotenoid-derived aroma compounds*. American Chemical Society, Washington DC, pp. 1-299. [10.1021/bk-2002-0802.ch001](http://dx.doi.org/10.1021/bk-2002-0802.ch001)

Wong, K. H., Abdul Aziz, S., & Mohamed, S. (2008). Sensory aroma from Maillard reaction of individual and combinations of amino acids with glucose in acidic conditions. *International Journal of Food Science & Technology*, *43*(9), 1512–1519. <https://doi.org/10.1111/j.1365-2621.2006.01445.x>

Wu, S., Lau, K. H., Cao, Q., Hamilton, J. P., Sun, H., Zhou, C., Eserman, L., Gemenet, D. C., Olukolu, B. A., Wang, H., Crisovan, E., Godden, G. T., Jiao, C., Wang, X., Kitavi, M., Manrique-Carpintero, N., Vaillancourt, B., Wiegert-Rininger, K., Yang, X., … Fei, Z. (2018). Genome sequences of two diploid wild relatives of cultivated sweetpotato reveal targets for genetic improvement. *Nature Communications*, *9*(1), 4580. <https://doi.org/10.1038/s41467-018-06983-8>

Xin, J., Huang, B., Dai, H., & Mu, Y. (2017). Characterization of root morphology and root-derived low molecular weight organic acids in two sweet potato cultivars exposed to cadmium. *Archives of Agronomy and Soil Science*, *63*(5), 723-734.

Xu, L., Yu, X., Li, M., Chen, J., & Wang, X. (2017). Monitoring oxidative stability and changes in key volatile compounds in edible oils during ambient storage through HS-SPME/GC–MS. *International Journal of Food Properties*, *20*(sup3), S2926–S2938. <https://doi.org/10.1080/10942912.2017.1382510>

Xu, M., Guo, J., Li, T., Zhang, C., Peng, X., Xing, K., & Qin, S. (2021). Antibiotic effects of volatiles produced by Bacillus tequilensis XK29 against the black spot disease caused by Ceratocystis fimbriata in postharvest sweet potato. *Journal of agricultural and food chemistry*, *69*(44), 13045-13054.

Yada, B., Brown-Guedira, G., Alajo, A., Ssemakula, G. N., Owusu-Mensah, E., Carey, E. E., Mwanga, R. O. M., & Yencho, G. C. (2017). Genetic analysis and association of simple sequence repeat markers with storage root yield, dry matter, starch and β-carotene content in sweetpotato. *Breeding Science*, *67*(2), 140–150. <https://doi.org/10.1270/jsbbs.16089>

Yan, M., Nie, H., Wang, Y., Wang, X., Jarret, R., Zhao, J., Wang, H., & Yang, J. (2022). Exploring and exploiting genetics and genomics for sweetpotato improvement: Status and perspectives. *Plant Communications*, *3*(5), 100332. <https://doi.org/10.1016/j.xplc.2022.100332>

Yang JinChu, Y. J., Wang HongWei, W. H., Li YaoGuang, L. Y., Liu Huan, L. H., Wang BaoLin, W. B., Hu ShaoDong, H. S., ... & Li HaoLiang, L. H. (2017). Effect of processing methods on sugar, aroma and sweetness of sweetpotato.

Yang, S., Lee, J., Lee, J., & Lee, J. (2007). Effects of riboflavin-photosensitization on the formation of volatiles in linoleic acid model systems with sodium azide or D2O. *Food Chemistry*, *105*(4), 1375–1381. <https://doi.org/10.1016/j.foodchem.2007.05.002>

Yao, Y., Zhang, R., Jia, R., Yao, Z., Qiao, Y., & Wang, Z. (2024). Exploration of Raw Pigmented-Fleshed Sweet Potatoes Volatile Organic Compounds and the Precursors. *Molecules*, *29*(3), 606. <https://doi.org/10.3390/molecules29030606>

Yavuzer, E., Özogul, F., & Özogul, Y. (2020). Impact of icing with potato, sweet potato, sugar beet, and red beet peel extract on the sensory, chemical, and microbiological changes of rainbow trout (Oncorhynchus mykiss) fillets stored at (3±1° C). *Aquaculture International*, *28*, 187-197.

Yaylayan, V. A. (2003a). Recent Advances in the Chemistry of Strecker Degradation and Amadori Rearrangement: Implications to Aroma and Color Formation. *Food Science and Technology Research*, *9*(1), 1–6. <https://doi.org/10.3136/fstr.9.1>

Yaylayan, V. A. (2003b). Recent Advances in the Chemistry of Strecker Degradation and Amadori Rearrangement: Implications to Aroma and Color Formation. *Food Science and Technology Research*, *9*(1), 1–6. <https://doi.org/10.3136/fstr.9.1>

Yaylayan, V. A., & Keyhani, A. (2001). Carbohydrate and Amino Acid Degradation Pathways in l -Methionine/ d -[^13^ C] Glucose Model Systems. *Journal of Agricultural and Food Chemistry*, *49*(2), 800–803. <https://doi.org/10.1021/jf000986w>

Yaylayan, V. A., & Mandeville, S. (1994). Stereochemical Control of Maltol Formation in Maillard Reaction. *Journal of Agricultural and Food Chemistry*, *42*(3), 771–775. <https://doi.org/10.1021/jf00039a034>

Yencho, G. C., Olukolu, B. A., & Isobe, S. (Eds.). (2025). *The Sweetpotato Genome*. Springer International Publishing. <https://doi.org/10.1007/978-3-031-65003-1>

Yencho, G. C., Pecota, K. V., Schultheis, J. R., VanEsbroeck, Z.-P., Holmes, G. J., Little, B. E., Thornton, A. C., & Truong, V.-D. (2008). ‘Covington’ Sweetpotato. *HortScience*, *43*(6), 1911–1914. <https://doi.org/10.21273/HORTSCI.43.6.1911>

Yooyongwech, S., Samphumphuang, T., Tisarum, R., Theerawitaya, C., & Cha-Um, S. (2016). Arbuscular mycorrhizal fungi (AMF) improved water deficit tolerance in two different sweet potato genotypes involves osmotic adjustments via soluble sugar and free proline. *Scientia Horticulturae*, *198*, 107-117.

Yooyongwech, S., Samphumphuang, T., Tisarum, R., Theerawitaya, C., & Cha-Um, S. (2017). Water-deficit tolerance in sweet potato [Ipomoea batatas (L.) Lam.] by foliar application of paclobutrazol: role of soluble sugar and free proline. *Frontiers in Plant Science*, *8*, 1400.

Zhang, R., Tang, C., Jiang, B., Mo, X., & Wang, Z. (2021). Optimization of HS-SPME for GC-MS Analysis and Its Application in Characterization of Volatile Compounds in Sweet Potato. *Molecules*, *26*(19), 5808. <https://doi.org/10.3390/molecules26195808>

Zhang, Z., & Li, G. (2010). A review of advances and new developments in the analysis of biological volatile organic compounds. *Microchemical Journal*, *95*(2), 127–139. <https://doi.org/10.1016/j.microc.2009.12.017>

Zhu, G., Gou, J., Klee, H., & Huang, S. (2019). Next-Gen Approaches to Flavor-Related Metabolism. *Annual Review of Plant Biology*, *70*(1), 187–212. <https://doi.org/10.1146/annurev-arplant-050718-100353>

Zhu, G., Wang, S., Huang, Z., Zhang, S., Liao, Q., Zhang, C., Lin, T., Qin, M., Peng, M., Yang, C., Cao, X., Han, X., Wang, X., Van Der Knaap, E., Zhang, Z., Cui, X., Klee, H., Fernie, A. R., Luo, J., & Huang, S. (2018). Rewiring of the Fruit Metabolome in Tomato Breeding. *Cell*, *172*(1–2), 249-261.e12. <https://doi.org/10.1016/j.cell.2017.12.019>

Zhu, L., Mu, T., Ma, M., Sun, H., & Zhao, G. (2022). Nutritional composition, antioxidant activity, volatile compounds, and stability properties of sweet potato residues fermented with selected lactic acid bacteria and bifidobacteria. *Food Chemistry*, *374*, 131500.
